# A structured and comprehensive review of Sino-Tibetan languages PoS taggers

**DOI:** 10.3389/frai.2026.1869572

**Published:** 2026-09-22

**Authors:** Bedawati Basumatary, Shikhar Kumar Sarma, Kuwali Talukdar, Mirzanur Rahman, Vaskar Deka, Parismita Sarma

**Affiliations:** Department of Information Technology, Gauhati University, Guwahati, Assam, India

**Keywords:** deep learning, hybrid model, low resource language, machine learning, PoS, Sino-Tibetan language

## Abstract

Parts of Speech (PoS) tagging is a fundamental activity in Natural Language Processing (NLP). It consists in assigning an appropriate grammatical category to each word in a text, such as noun, verb, adjective or adverb. It serves as a crucial preprocessing step in many NLP applications such as machine translation, information retrieval, text summarization and sentiment analysis. PoS tagging has been significantly improved, however, obtaining high accuracy for different languages is still an active research challenge. Researchers have developed different ways throughout the years starting from simple lexical methods to rule-based and statistical techniques and most recently hybrid, deep learning and transformer-based models which provide better performance. This study reviews the current PoS tagging approaches comprehensively and discusses the various strategies, its advantages and limitations. Special emphasis is made on languages of the Sino-Tibetan family, many of which are considered as low-resource, due to the lack of annotated corpora and linguistic resources. As shown by the survey, relatively little work has been done on PoS tagging for these languages compared to the far more extensively studied languages. To fill this gap, this study examines existing studies on PoS tagging of several Sino-Tibetan languages, explains the datasets and models used, and compares their stated performance. Furthermore, the article discusses the limits of the prior approaches and highlights the major research gaps and future research objectives to create more robust machine learning, deep learning, hybrid and transformer-based PoS tagging systems for low-resource Sino-Tibetan languages.

## 1 Introduction

As applications of Artificial Intelligence (AI) expand daily in all areas of life, natural language processing, or NLP, is becoming more and more significant. With almost 400 languages, the Sino-Tibetan language family is one of the largest language families in the world. The two main branches of the Sino-Tibetan language family are the Tibeto-Burman languages, which are spoken throughout the Tibetan plateau, the Himalayas, and portions of Southeast Asia, and the Sinitic languages, which are spoken mostly in China and include Mandarin, Cantonese, and others. Given the advancements in technology and the growing efforts to create resources and tools for underrepresented languages, there is definitely a trend toward more inclusion and exploration of these languages in NLP research, especially because the majority of these languages already have a user audience. One of the key components in the creation of any significant application in the fields of natural language processing in the contemporary world is the Part of Speech (PoS) Tagger. The PoS tags that are annotated with the sentence tokens are one significant embellishment. Automatic tagging involves a model that examines the token and then assigns a PoS tag to it based on certain assumptions, decision-weights, or learning, whereas linguistic tagging involves the manual assignment of lexical categories to the individual tokens. Each token in an input sentence is given a distinct part of speech tag, such as noun, verb, adjective, or adverb, by the PoS tagger. It is one of the fundamental technologies required for many NLP applications, including- Word Sense Disambiguation (WSD), Information Processing (IP), Machine Translation (MT), and Information Retrieval (IR). Researchers have been attempting to use Machine Learning (ML) and Deep Learning (DL) techniques to meet the need for effective PoS taggers. In the discipline of NLP, these methods are becoming more well-known and acknowledged. Both ML and DL are used to annotate the words from the complex pattern with the appropriate PoS and to extract hidden features and important information from criteria defined by linguists. Dictionaries and grammatical rules were employed to determine the tag of each word. This approach is effective for limited datasets, but it fails when dealing with new and unfamiliar words. Afterwards, researchers commenced the utilization of machine learning models. Hidden Markov Models (HMM), Conditional Random Fields (CRF), and Support Vector Machines (SVM) gained prominence. These models acquire patterns from labeled text. They yield superior outcomes compared to rule-based models, but require extensive labeled corpora for optimal performance. Later, deep learning was used for PoS tagging using neural networks such Convolutional Neural Networks (CNN), Bidirectional LSTM (BiLSTM), and Recurrent Neural Networks (RNNs). These models automatically learn the meaning of words (embeddings) and contextual information. They minimize the requirement for manual feature design. However, they also require more training data. Transformer models such as BERT (Bidirectional Encoder Representations from Transformers), mBERT (Multilingual Bidirectional Encoder Representations from Transformers), XLM-R, and Muril are used in the most modern techniques. These models are pre-trained on vast amounts of multilingual data and can be finetuned for languages with limited resources. They provide state-of-the-art results and are especially effective for Sino-Tibetan languages with limited corpora. The several Machine Learning (ML) and Deep Learning (DL) techniques utilized in PoS tagging for Sino-Tibetan languages were described in the article. Additionally, this has offered a thorough analysis of the current methods used by PoS taggers. Highlighted are the methods employed and their performances. Research gaps and upcoming projects are finally covered.

The subsequent sections of this study are structured as follows:

- Section 2 provides an overview of Sino-Tibetan languages, emphasizing their linguistic features and implications for natural language processing applications.
- Section 3 describes the search methods employed for the review.
- Section 4 discusses recent advancements in NLP research for selected low-resource languages.
- Section 5 provides a comprehensive review of PoS tagging approaches for Sino-Tibetan languages, including language-wise analysis of Myanmar, Manipuri, Mizo, Kokborok, Chinese, Nyishi, Bodo, Tibetan, and Tenyidie.
- Section 6 provides an analysis of the dataset.
- Section 7 addresses the performance evaluation measures typically employed for PoS taggers.
- Section 8 presents a comparative analysis of PoS tagging strategies, encompassing rule-based, statistical, machine learning, deep learning, transformer-based, and hybrid approaches.
- Section 9 presents an analysis of the reviewed studies, limitations of the review, and research gap and future directions.
- Finally, Section 10 concludes the study.

## 2 Overview of Sino-Tibetan languages: linguistic features and NLP implications

With more than 400 languages spoken by more than a billion people, the Sino-Tibetan language family is one of the largest language groups in the world. It can be broadly classified into two branches (LaPolla and Thurgood, 2006): the Tibeto-Burman branch, which contains a variety of languages spoken in India, Nepal, Bhutan, Myanmar, and neighboring areas, and the Sinitic branch, which includes Chinese languages like Mandarin, Cantonese, and Wu. In the context of this review, both Sinitic (Chinese) and non-Sinitic, Tibeto-Burman languages are considered. While Chinese represents a high-resource branch with mature PoS tagging systems, non-Sinitic Sino-Tibetan languages are generally low-resource and underrepresented in NLP research. Including both branches allows a comparative understanding of how resource availability and linguistic diversity influence PoS tagging performance across the Sino-Tibetan family. A number of Sino-Tibetan languages, such as Tibetan, Manipuri (Meitei), Bodo, Mizo, Nyishi, and Burmese, have been reported in the literature for PoS tagger. Different scripts, including Tibetan, Meitei Mayek, Devanagari, Roman, and Burmese scripts, are used to write these languages. The variety of writing systems offers obstacles for developing standardized NLP tools and resources, since each script frequently requires language-specific preprocessing, tokenization, text normalization, and annotation procedures. Many of the Sino-Tibetan languages also have sophisticated linguistic features. Rich morphology, agglutinative word formation, extensive use of prefixes and suffixes, conjunct characters and orthographic variations. Moreover, words often have a range of grammatical properties (tense, aspect, case, number, etc.) encoded within the same lexical item. These features add to the complexity of language processing tasks such as tokenization, feature extraction, PoS tagging, parsing and machine translation. Another important feature of several Sino-Tibetan languages is a relatively variable word order which makes context modeling and syntactic analysis more difficult. Additionally, function words and grammatical markers can exist as affixes or clitics rather than as independent tokens, which complicates segmentation and tagging. Lexical ambiguity is also widespread, where the same surface form might belong to various grammatical categories depending on the context. In addition, the absence of standard PoS tagsets and annotation guidelines further hinders study on many Sino-Tibetan languages. As a consequence, researchers generally work using language-specific tagsets and annotation techniques, which make cross-language comparison and benchmarking challenging. Furthermore, most of the Sino-Tibetan languages are low-resourced, e.g., lack of large-scale annotated corpora, limited digitized text, and no linguistic tools, e.g., morphological analyzers and parsers (Liu and Best, 2025). These limitations hinder the use of data heavy methodologies and stimulate the use of hybrid, transfer-learning and multilingual strategies.

Recent studies have shown the promise of cross-lingual transfer learning and multilingual pre-trained models to mitigate the data scarcity for the Sino-Tibetan languages. However, their performance differs in different languages because of the variances in writing systems and domain characteristics. The distinctive orthographic and lexical patterns restrict the transferability of representations learned from high-resource languages, while the domain mismatches in text genre, topic and writing style might negatively impact model performance and generalization. Therefore, it is crucial to understand the linguistic and resource characteristics of the Sino-Tibetan languages in order to evaluate the present PoS tagging systems and direct the future research in this area.

## 3 Search methodology

A systematic literature search was conducted to find Sino-Tibetan PoS tagging research. Academic databases like Google Scholar, IEEE Xplore, ACM Digital Library, SpringerLink, Scopus, ScienceDirect, ACL Anthology, and Semantic Scholar were searched. These databases were chosen for their extensive NLP, Computational Linguistics, AI, and Language Technology research coverage. The search included 2008–2026 publications. To find relevant studies, keywords and Boolean operators were used. The top search terms were “Part of-Speech Tagging,” “PoS Tagging,” “PoS Tagger,” “Natural Language Processing,” “Sino-Tibetan Languages,” and “Low-Resource Languages.” The search queries included language-specific keywords including “Bodo,” “Manipuri,” “Mizo,” “Tibetan,” “Myanmar,” “Chinese,” “Kokborok,” and “Nyishi.” The search expressions were tailored to database search functions. The aim of this review was to study the research done on PoS tagging for Sino-Tibetan languages. In Section II some NLP studies on languages such as Bodo, Assamese and Manipuri are briefly discussed to give an idea of the overall progress of NLP research in low-resource languages, but these studies have not been included in the systematic review process if they did not directly address PoS tagging or the development of linguistic resources directly contributing to PoS tagger construction. The retrieved records were checked in several steps. The duplicates were deleted at the beginning. Then, the titles and abstracts were screened for research related to PoS tagging in Sino-Tibetan languages. Studies on other NLP tasks, like machine translation, named entity recognition, text summarization, sentiment analysis, word sense disambiguation, transliteration, speech processing and associated applications were excluded. The remaining studies were then screened for eligibility in full-text. The inclusion criteria were (i) studies on PoS tagging for Sino-Tibetan languages, (ii) studies that propose a PoS tagging methodology or report experimental results, or (iii) studies on the development of basic linguistic resources such as annotated corpora, tagsets, dictionaries and lexical resources that directly support PoS tagger development. For several extremely low-resource Sino-Tibetan languages like Mizo and Nyishi, where the work on PoS tagging is limited, it was felt that resource creation studies need to be included. We rejected studies on other than Sino-Tibetan languages, non relevent NLP tasks, duplicates and ones without sufficient methodological and experimental details. From each selected study we retrieved and examined language, dataset characteristics, tagset design, tagging process, machine learning or deep learning techniques, evaluation metrics and performance reported. The PRISMA flow diagram summarizes the entire process of study identification, screening, eligibility assessment and final inclusion in Figure 1.

**Figure 1:**
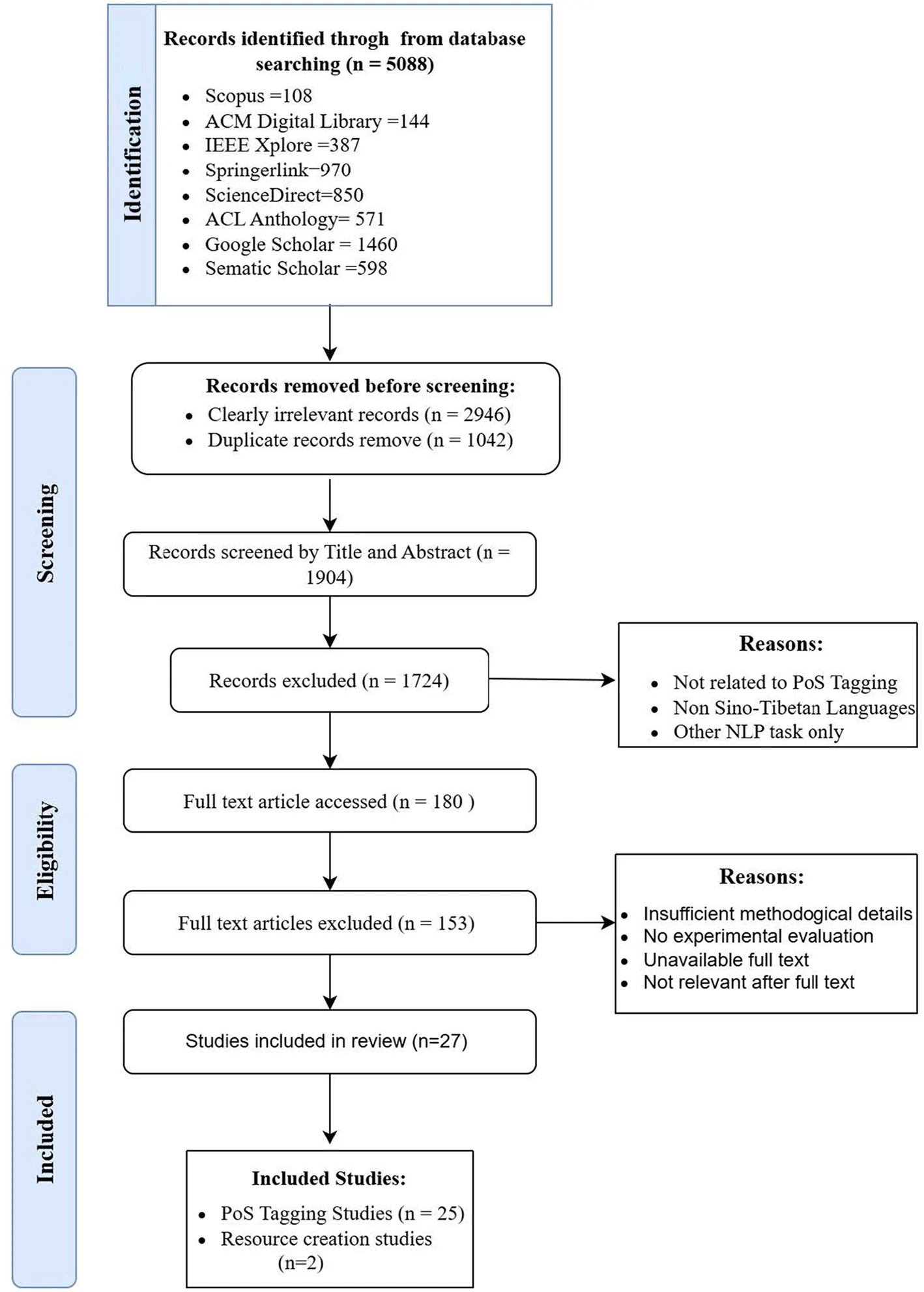
PRISMA flow diagram of the literature screening process.

## 4 Advancement of NLP works in few related low resource languages

In the field of natural language processing (NLP), relatively less research work has been done on languages such as Bodo and Assamese. Bodo is a Sino-Tibetan language, and both Bodo and Assamese are major languages in Assam, the northeastern state of India. We concentrate on these two low resource languages, and target different NLP works. Multilingual NLP works in these languages also included other low resource languages of North East India, such as Manipuri. There are collaborative works in between educational institutes involving Assamese, Bodo, Manipuri languages. These includes multilingual Machine Translation between pairs of these languages. These languages are spoken primarily in the northeastern region of India. Here we mention few of these works, as they are closely related in terms of geographical coverage, as well as multilingual NLP works. The work in the field of NLP for Bodo language is comparatively lesser than Assamese language. Digital content for both the languages is very less compared to other Indian languages. Preliminary works could be traced using different ML and DL techniques. Neural Machine Translation work involving Bodo has been conducted by Talukdar et al. (2023). PoS tagging is crucial and fundamental work in the NLP field, and structured reviews for Indo Aryan family languages have been compiled (Talukdar and Sarma, 2023a). Work such as DL based Bodo PoS tagger has also been started (Basumatary et al., 2023). While surveying in Assamese language we have encountered works such as LSTM and BiLSTM based PoS tagger for Assamese Language (Talukdar and Sarma, 2023b) where Universal PoS tagset has been used for efficient tagger design. Assamese Word Sense Disambiguation (WSD) research has also been undertaken by various groups. A comprehensive WSD review (Sarmah and Sarma, 2016a) presents details about past works on Indo Aryan languages WSD. Application oriented local language NLP work like prediction and correction of words using syntactic analysis has been presented [26] Similar application-based works using WordNet has also been found (Barman et al., 2013; Sarma et al., 2012). Bodo language NLP is with very low resources, yet few works could be traced, like the design and development of Bodo Wordnet (Sarma et al., 2010). Low resource NLP is really a challenge, and various techniques and applications have been tried to work with, like lemmatizer, WSD using genetic algorithm (Gogoi and Baruah, 2022; Gogoi et al., 2020). Another interesting work has been the WSD with Cuckoo Search Algorithm (Gogoi et al., 2021). Corpus statistics have also been used for efficient design of WSD for low resource language (Sarmah and Sarma, 2016b; Baruah et al., 2021a). Wordnets have been used for efficient NLP pipeline design like summarization (Baruah et al., 2019). Name Entity Recognition (NER) (Gayary et al., 2025), corpus creation, Machine translation (Baruah et al., 2014; Ahmed M. A. et al., 2023; Ahmed M. et al., 2023; Kashyap et al., 2024), Text Summarization using Deep learning (Goutom et al., 2023), Graph based extractive text summarization (Baruah et al., 2022), Hybrid based extractive text summarization (Baruah et al., 2020). summarization using statistical features of Wordnet (Sarmah et al., 2019; Baruah et al., 2021b; Moirangthem et al., 2022), Hate Speech Detection (Baruah et al., 2023b,a), Spelling Error Detection (Phukan et al., 2023), Toxic Comment Detection (Neog and Baruah, 2023) etc have already been accomplished by different researchers and research groups. Due to lack of resources, NLP work in the Bodo language is still lagging behind (Brahma et al., 2012). Another language like Manipuri, is also a low resource language, belonging to the Sino-Tibetan family. This language is also slowly increasing in the field of NLP. Some works done in Manipuri are Corpus Creation (Moirangthem et al., 2022), NER (Jimmy et al., 2023; Htike et al., 2017), Transliteration based Manipuri (Moirangthem and Nongmeikapam, 2021; Nongmeikapam et al., 2012b), MultiWord Named Entities (MNEs), etc. (Laishram et al., 2020). Other NLP research works on Low resource Manipuri language includes the effect of Multiword Expressions (MWEs) in efficient PoS tagger design (Nongmeikapam et al., 2012d,a), Genetic Algorithm based MWE detection modeling (Nongmeikapam and Bandyopadhyay, 2011) CRF based modeling of MWE detection (Nongmeikapam et al., 2011; Nongmeikapam and Bandyopadhyay, 2010), Incremental Model building with PoS and Reduplicated MWE (Nongmeikapam et al., 2014), Automatic Segmentation (Nongmeikapam et al., 2012c) etc. This study surveyed and tried to accumulate knowledge regarding other languages of the same family such as Myanmar, Manipuri, Mizo, Bodo, Kokborok, Tibetan, Chinese.

## 5 PoS tagger for Sino-Tibetan languages

### 5.1 Myanmar language

#### 5.1.1 Rule based approaches

The study Minn and Soe (2019) on Myanmar language NLP explored word morphology, implemented n-gram based word segmentation, and proposed Rule Based stemming and PoS tagging. The system processes Myanmar words by removing prefixes, infixes, and suffixes for segmentation and tagging. Using 300 sentences from Wikipedia, the MCF (Myanmar ComputerFederation) dataset, and a Myanmar Grammar Book, the model achieved an F1-score of 86.16% for segmentation, 81.46% for stemming, and 78.54% for PoS tagging, with an overall accuracy of 80%.

#### 5.1.2 Statistical approaches

The Kayah language is spoken by the Kayah Li community in Myanmar. The work presents a novel evaluation of PoS tagging, especially designed for the Kayah Language, a low- resource language in Myanmar, is presented by Linn and Patil (2019). The study uses a Kayah corpus translated by native speakers and proposes designing an HMM based PoS tagger model. For this work, sixteen tags were constructed and trained on a corpus comprising a total of 3,994 words using the HMM model. This comprehensive training improves robust linguistic analysis and tagging accuracy. The model is trained using annotated data and then applied to decode using the Viterbi algorithm, which determines the most probable sequence of PoS tags for a given sentence. The experimental results demonstrate promising performance, with an overall f1_score of 87% achieved on the test data, showcasing the effectiveness of the Kayah HMM-based PoS tagging approach. A work Lam and Soe (2020) presents a Myanmar language corpus for combined word segmentation and PoS tagging. With 12 PoS categories assigned to it, the corpus consists of 839,161 words and 51,207 sentences. This data was used to train and test a HMM, which demonstrated its effectiveness for Myanmar NLP applications with an accuracy of 94%.

#### 5.1.3 Hybrid approaches

The authors Myint (2011) designed a novel hybrid PoS tagging system for Burmese (Myanmar) texts employing both rule-based and probabilistic methods. The model initially segmented sentences using predefined rules, then assigned tags by combination of rule-based methods and CRF based approach for PoS disambiguation. It integrated Myanmar specific tagsets, segmentation rules, and CRF tagging algorithms. The proposed system was tested on the UCSM (University of Computer Studies, Mandalay) lexicon using 400 sentences (5,225 tokens) with 88 grammatical tags, achieving an average accuracy of 84.71%, 89.53% for known words and 79.89for unknown words.

#### 5.1.4 Result analysis

The research on Myanmar PoS tagging is still limited, with only a handful of studies that address rule-based, statistical, and hybrid approaches. The earliest work achieved an accuracy of 84.71% by employing a hybrid rule-based and CRF model. The challenges of managing linguistic ambiguity and unseen terms through handcrafted rules alone were reflected in the lower F1-score of 78.54% reported by pure rule-based methods. The highest reported performance was attained by statistical approaches, particularly HMM-based models, which achieved an accuracy of 94%. Despite the fact that the hybrid approach outperformed the pure rule-based system, its performance was lower than that of the statistical model. This may be attributed to the use of a larger tagset, limited training data, and challenges in treating unknown terms. In general, the findings indicate that Myanmar PoS labeling is generally more effective when using data-driven approaches. Nevertheless, it is important to exercise caution when comparing studies directly due to the variations in datasets, corpus sizes, tagsets, and evaluation methodologies.

### 5.2 Manipuri language

#### 5.2.1 Rule based approaches

A rule-based PoS tagger was developed for the highly agglutinative Manipuri language (Singha et al., 2012a) using ILPOST (PoS Tagset for Indian languages) tagset of 97 tags and hand-written linguistic rules for affix stripping technique to segment roots from affixes. Assigning morpho-syntactic categories is challenging due to limited PoS tagged corpora. The tagger, tested on word sets of 100, 500, and 1000, progressively yielding accuracy from 50% to 85%. This tool is expected to enhance PoS analysis for the Manipuri language. A study Singh and Bandyopadhyay (2008) described the development of a morphology-driven PoS tagger for Manipuri, which addressed the lack of PoS tagged corpora by utilizing three dictionaries for root words, prefixes, and suffixes. This technique tags words according to affix information rather than context. They used a Manipuri tagset with 13 PoS tags. When tested on 3,784 sentences with 10,917 unique words, the system obtained 69% accuracy. Errors were largely due to unknown words (23%), including 9% named entities, and 8% incorrect tags for known words. The research highlights the potential of morphology-based tagging for Manipuri and suggests improvements for handling unknown words and named entities.

#### 5.2.2 Statistical approaches

A work Singha et al. (2012b), designed an HMM-based statistical tagger using a 2,000 tagged word corpus from a rule-based Manipuri PoS tagger (Singha et al., 2012a). The performance's tagging system is evaluated on 97 morphosyntactic categories, Despite only 12 tags being used in the statistical analysis, the system attained 92% accuracy, with 80% of automatically generated sequences matching the manually tagged output.

#### 5.2.3 Machine learning approaches

Singh (2008) developed a ML based PoS Tagger for Manipuri language, using CRF and SVM techniques on a manually annotated dataset of 63,200 tokens with 26 PoS tags. Training and testing were conducted on 39,449 and 8,672 tokens, respectively. The taggers achieved accuracy of 72.04% (CRF) and 74.38% (SVM). Another study Nongmeikapam et al. (2010) used an unsupervised CRF based approach for PoS tagging in Manipuri, utilizing features like neighboring words, PoS tags, prefixes, suffixes, and word length to address the language's agglutinative nature. A dataset of 40,000 manually tagged words was used, with 35,000 for training and 5,000 for testing. The system achieved 70.00% recall, 77.78% precision, and 73.68% f1 score, highlighting CRF's effectiveness. Despite the promising results, further research is needed to explore limitations and improve accuracy.

#### 5.2.4 Result analysis

The evaluated works show Manipuri PoS tagging research progressing from rule-based to statistical and machine learning methods. Early rule-based systems used linguistic information, morphological analysis, and affix stripping to handle the language's agglutination. These approaches showed the importance of language-specific linguistic resources, but manual rule generation and unseen word processing limited their efficacy. Statistics and machine learning allowed contextual patterns to be learned from annotated corpora without handwritten rules. These methods were better at capturing contextual information and resolving lexical ambiguities in Manipuri, which has rich morphology and complex word building. However, annotated data, feature engineering techniques, and language resources affect these approaches' efficacy. Unstandardized datasets and tagsets plagued the reviewed studies. Multiple research used different corpus sizes and tagset designs, making comparisons difficult. PoS tagging is also hindered by Manipuri's agglutinative structure, many affixes, and unfamiliar terms. The findings indicate that contextual statistics and machine learning methods are better for Manipuri PoS tagging than rule-based methods. However, the lack of huge annotated corpora limits future study, requiring standardized resources and improved deep learning and transformer-based methods.

### 5.3 Mizo language

#### 5.3.1 Resource creation

The study Pakray et al. (2015) developed linguistic resources and a PoS tagger for the under-resourced Mizo language, also known as Lushai, from the Kukish branch of the Sino-Tibetan family. A Mizo-to-English dictionary with 26,407 words was created using both automated and manual methods. Additionally, a 24-item PoS tag set was developed based on the study of Mizo grammar. These resources aim to support the creation of an automated PoS tagger, contributing to the development of NLP tools for the Mizo language.

#### 5.3.2 Machine learning approach

Another study Nunsanga et al. (2021) created a PoS tagger for the Mizo language that used a CRF model to assign PoS tags to tokens in sentences. A manually annotated corpus of approximately 30,000 words and a total of 41 PoS tags were utilized to train and evaluate the system. The tagger scored 89.46% accuracy, 89.3% F1-score, 89.42% precision, and 89.48% recall, indicating that the CRF model is effective for PoS tagging in the Mizo language.

#### 5.3.3 Hybrid approach

The study Nunsanga et al. (2023) combined HMM, N-gram taggers, and rule-based methods to create a hybrid PoS tagging framework for the low-resource Mizo language. The study manually annotated 72,077 Mizo tokens and created a 48-PoS tagset. Before improving the baseline HMM tagger, the authors improved Viterbi decoding to handle unfamiliar words. HMM, N-gram tagging, and Mizo-specific morphological rules were then combined in a hybrid architecture. Experimental results showed that the hybrid model outperformed the baseline HMM and other N-gram models with 84.29% accuracy. Weighted average precision, recall, and F1-scores of 83.09%, 77.88%, and 79.64% showed that statistical and rule-based techniques for Mizo PoS tagging work.

#### 5.3.4 Result analysis

According to the studied papers, Mizo language PoS tagged research has moved from resource development to statistical and hybrid tagging. Language resources including annotated corpora, dictionaries, and tagsets have helped develop automatic PoS taggers. The CRF-based model performed best, indicating that machine learning can capture contextual information and resolve tagging ambiguities in Mizo literature. The hybrid HMM-based technique, which included statistical and rule-based methodologies, performed well for unknown words and language-specific morphological patterns. Since the research used varied corpus sizes, tagset designs, and evaluation conditions, direct comparison of stated performances should be done cautiously. The findings indicate that data-driven methods outperform standard statistical methods, whereas hybrid methods are useful for low-resource languages with limited annotated data. Larger standardized corpora and contemporary deep learning and transformer-based architectures may increase Mizo PoS tagging performance in future study.

### 5.4 Kokborok language

#### 5.4.1 Hybrid approaches

The work Patra et al. (2012) describes the creation of a PoS tagger for the under-resourced Kokborok language utilizing both rule-based and supervised methodologies. A rule-based approach utilizing a morphological analyzer attained 70% accuracy on 42,537 words across 26 PoS categories. The supervised methodology assessed CRF and SVM classifiers using a dataset of roughly 40,000 words. The CRF model, integrating contextual and morphological information, attained an accuracy of 81.67%, whilst the SVM classifier reached the greatest accuracy of 84.46%. The findings demonstrate that supervised learning techniques surpass merely rule-based methods in their ability to capture contextual dependencies and address lexical ambiguities. The performance enhancement realized by integrating contextual, affix, and morphological information underscores the significance of linguistic aspects in processing morphologically rich and low-resource languages like Kokborok. The architecture of the proposed SVM model is illustrated in Figure 2. The framework incorporates contextual and morphological elements to efficiently capture linguistic patterns and increase the tagging accuracy. Nonetheless, despite the encouraging outcomes, the overall accuracy is inferior to that documented for other resource rich languages, likely due to the restricted size of the annotated corpus and the lack of standardized linguistic resources. The lack of benchmark datasets hinders direct comparison of the proposed approach's efficacy with other PoS tagging systems. Nonetheless, the study establishes a robust foundation for Kokborok PoS tagging and illustrates that the incorporation of machine learning methodologies with language-specific linguistic insights can significantly enhance tagging efficacy.

**Figure 2:**
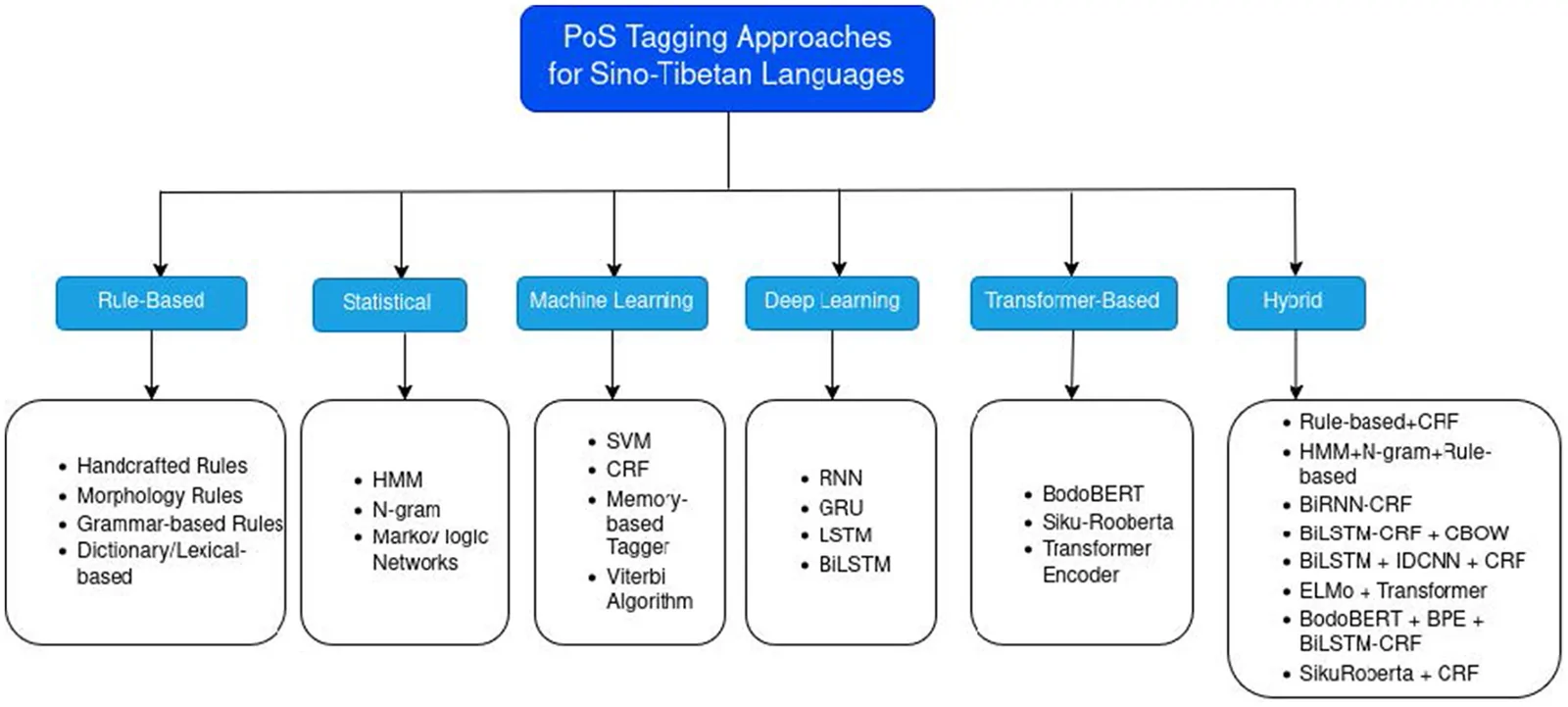
Proposed taxonomy of PoS tagging approaches for Sino-Tibetan languages based on the reviewed literature.

### 5.5 Chinese language

#### 5.5.1 Statistical approaches

A Markov Logic Networks (MLNs) based Chinese PoS Tagging method has been developed in study Liao et al. (2015). They first created two comparable datasets representing both short and long sentences. 14,610 characters or 4,240 words are included in dataset 1, and 57,440 characters or 8,377 words are included in dataset 2. Both datasets are segmented into words with open source software ICTCLAS (Zhang et al., 2003), manually processed for PoS tags, and annotated with pinyin (phonetic notation) and PoS tags. For two-fold cross-validation, Dataset 1 is split evenly between training and testing subsets, each containing 13,910 and 12,810 predicates. For three-fold cross-validation, Dataset 2 is divided into three sections with 29,940, 34,220, and 31,270 predicates. After that, the PoS tags and annotated words are transformed into MLN predicates. The system achieved 87.5% of MP (Matched Precision), 84.9% of MR (Matched Recall) and 86.2% of F1-score on datasets 1 and 88.2% of MP, 83.5% of MR and 85.8% of F1-score on Datasets 2.

#### 5.5.2 Deep learning approach

The study Shen et al. (2022) introduced a DL framework for low-resource Ancient Chinese word segmentation and PoS tagging with a Roberta-CRF architecture based on the pre-trained SikuRoberta model. The research utilized a hybrid tagset of 22 PoS tags alongside segmentation labels and implemented data augmentation methods utilizing LSTM language models and Masked Language Models (MLMs) to mitigate data scarcity. Experimental results validated the efficacy of the suggested methodology, attaining F1-scores of 93.92% for word segmentation and 88.12% for PoS tagging on the in-domain test set, while achieving 87.53% and 78.31%, respectively, on the out-domain test set. To address the lack of annotated corpora, the researchers (Feng and Li, 2023) suggested a distant supervision system based on data augmentation approaches for low-resource Ancient Chinese PoS tagging. The study created weakly labeled Ancient Chinese data from a parallel corpus of 967,257 Modern Chinese-Ancient Chinese phrase pairs using word alignment and annotation projection. A SikuRoberta-CRF architecture was used with a hybrid tagset of 22 PoS tags and four segmentation tags. To mitigate the labeling noise caused by distant supervision, the authors designed a relabeling technique on a manually annotated corpus of 8,701 sentences. We assessed the proposed model on an in-domain dataset Test-A (1,594 sentences from Zuozhuan) and an out-of-domain dataset Test-B (2,150 sentences from Shiji and Zizhitongjian). Experimental findings demonstrated the success of the method with WSG and PoS F1-scores of 95.64% and 90.55% on Test-A, and 93.64% and 86.21% on Test-B, respectively, outperforming numerous existing data augmentation approaches for Ancient Chinese PoS tagging. Another deep learning-based study with a data-driven model was proposed by the authors Xu and Wang (2022) for automated Chinese word segmentation and PoS tagging. In this system, text representation was done using Word2Vec, feature extraction was performed using an upgraded AlexNet network with attention mechanism, contextual learning was carried out using a neural network language model, and sequence labeling was implemented with a CRF layer. We assessed the model using three public Chinese datasets, MSR, CTB7 and CTB5, which include 494K, 78K and 2,131K training words respectively. Experimental results show that the proposed model outperforms the baseline models (Bi-LSTM, CNN and BiRNN) in terms of the Micro F1-score and Macro F1-score over all the evaluated datasets, and also improves the training efficiency, which confirms the effectiveness of the proposed DL architecture for Chinese word segmentation and PoS tagging.

#### 5.5.3 Hybrid approach

Using a Bidirectional RNN-CRF architecture, the work Shao et al. (2017) presents a character-based model for combined segmentation and PoS tagging in Chinese. To improve tagging accuracy, the model used vector representations that capture contextual and sub character characteristics of Chinese characters. Character embeddings are trained on the Sogou News Corpora (SogouCS) and Wikipedia using the GloVe embedding model. Randomly initialized vectors are used to represent characters that are not included in the embedding vocabulary. However, for higher-order n-grams, the model does not employ pre-trained embeddings. The proposed model performs well across a range of dataset sizes, genres, and annotation techniques; Chinese Treebank 5.0 (CBT5) with approximately 494k training words, Chinese Treebank 9.0 (CBT9) with about 1.7M training words and Universal Dependencies (UD) Chinese with around 99k training words and higher OOV rates. When tested on the CTB5, CTB9, and UD Chinese datasets (Xue et al., 2005; Nivre et al., 2016). With a 94.38 F1-score, it notably obtains state-of-the-art performance on the CTB5 dataset which is around 8k testing word, highlighting its accuracy and resilience for processing Chinese.

#### 5.5.4 Result analysis

The explored articles show that Chinese PoS tagging research has progressed from statistical methods to deep learning and hybrid architectures. On tiny annotated datasets, the MLN-based statistical model performed competitively, proving that probabilistic approaches can capture Chinese linguistic dependencies. Their manual feature engineering and limited contextual modeling limit their scalability and adaptation to vast and diverse corpora. Recent studies have shown that DL techniques automatically learn contextual and semantic representations from data, improving PoS tagger performance. Roberta-CRF and SikuRoberta-CRF architectures performed well for Ancient Chinese PoS tagging, showing that pre-trained language models, distant supervision, and data augmentation may handle the lack of annotated historical corpora. However, the performance disparity between in-domain and out-of-domain datasets implies that domain variation remains a problem even with advanced neural architectures. Using neural representation learning with sequence-labeling frameworks has also been successful. The character-based BiRNN-CRF model has one of the best F1-scores (94.38%), demonstrating the benefits of contextual character representations and CRF-based structured prediction. Attention-enhanced DL models increased segmentation and tagging across several benchmark datasets, proving the importance of contextual feature learning and long-range dependency modeling. Direct comparison between published studies is challenging due to differences in dataset size, annotation techniques, language types (Modern vs. Ancient Chinese), and evaluation protocols. In general, DL and hybrid architectures beat classic statistical methods, while pre-trained language models, contextual embeddings, and sequence-labeling frameworks are the best Chinese PoS tagging methods.

### 5.6 Nyishi language

#### 5.6.1 Resource creation

The paper Siram et al. (2022a) explores the creation of linguistic resources for PoS tagging in the Nyishi language, which addresses issues such as word identification. The authors used both human and automatic approaches to construct a 36-item tag set and 25,000 words from Nyishi to English dictionary. They explain the dictionary creation process and suggest an automatic PoS tagger for Nyishi. The report also discusses obstacles such as ambiguity, foreign words, and orthographic issues, which highlight the complexities of generating Nyishi language resources.

#### 5.6.2 Statistical approaches

This research Siram et al. (2022b) focuses on Nyishi PoS tagging utilizing a first-order, fully connected HMM. A corpus including around 30,000 Nyishi characters is employed, with the Viterbi technique implemented for word segmentation. The system attains 89% tagging accuracy by employing a Penn Treebank inspired tag set and disambiguation algorithms.

#### 5.6.3 Result analysis

For Nyishi Language, only two PoS tagging studies have been in the literature. One study mainly concentrates on resource building, which establishes the fundamental framework for further computational research. This includes corpus construction and tagset design. In the second study, a statistical method for PoS tagging is experimentally implemented. Despite the scarcity of annotated data, the experimental study shows that statistical techniques are workable for Nyishi which is achieved 89% of Accuracy. However, the result shows that more sophisticated models and larger datasets are required because of the limited resources and small corpus size. Overall, PoS tagging research for Nyishi is still in its early stages and needs more investigation employing hybrid and DL techniques, even though preliminary attempts yield encouraging findings.

### 5.7 Bodo language

#### 5.7.1 Deep learning approach

The paper Basumatary et al. (2023) concentrates on PoS tagging for the Bodo language, employing DL techniques like RNN, LSTM, GRU, and BiLSTM. A corpus of 9,991 sentences was annotated using 36 BIS tags. Extensive trials were carried out to evaluate these models, with the BiLSTM model achieving the best accuracy of over 98%. This represents a huge step forward for Bodo's PoS tagging, giving a useful tool for linguistic analysis and improving NLP resources for low resource languages.

#### 5.7.2 Hybrid approach

The paper Pathak et al. (2025) describes two significant advances for the Bodo language in NLP: the creation of BodoBERT, a specialized language model, and an ensemble PoS tagging model. This PoS tagger uses BiLSTM with CRF, as well as stacking BodoBERT embeddings with BytePairEmbeddings, to improve accuracy and resilience. The top performing model had an outstanding F1_score of 0.8041. The paper also contains a comparison examination of PoS taggers for Assamese, which highlights linguistic similarities between the two languages and improves the evaluation of PoS tagging performance in related languages.

#### 5.7.3 Result analysis

The analyzed works illustrate the progression of Bodo PoS tagging from traditional DL models to more sophisticated hybrid architectures. The previous study provided a preliminary benchmark for Bodo PoS tagging utilizing recurrent neural network methodologies and underscored the promise of deep learning techniques for the language. The subsequent article presented a more advanced system by using BodoBERT, BytePairEmbeddings, BiLSTM, and CRF, indicating a transition toward pre-trained language models and sequence-labeling architectures. The study conducted a comprehensive evaluation using tag-wise analysis, confusion matrices, and error analysis, yielding profound insights about the difficulties of PoS tagging in Bodo. The results demonstrate that linguistic ambiguity, data scarcity, and class imbalance continue to pose substantial obstacles, especially for infrequent part-of-speech categories. The transition from traditional deep learning models to hybrid architectures illustrates the advancing sophistication of Bodo PoS tagging research and highlights the significance of language-specific resources and pre-trained models in enhancing NLP applications for low-resource languages. Figure 3 depicts the architecture of the proposed PoS tagging framework based on BiLSTM for Bodo language.

**Figure 3:**
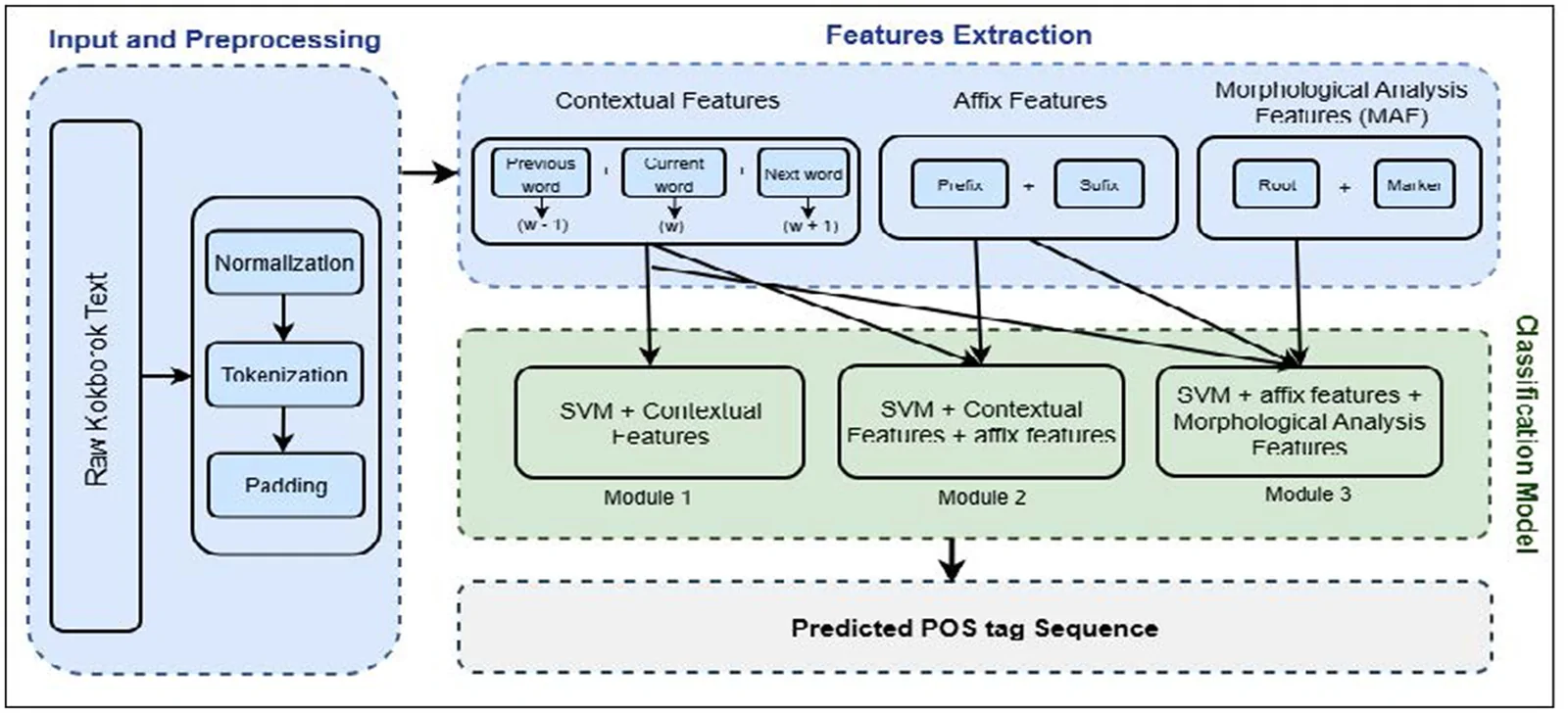
Architecture of SVM based PoS tagger system for the Kokborok language, adapted from Patra et al. (2012).

### 5.8 Tibetan language

#### 5.8.1 Machine learning approach

The study Meelen and Hill (2017) presents a novel approach to word segmentation and PoS tagging in Classical Tibetan that employs a Memory-Based Tagger (MBT). The suggested workflow consists of three stages: memory-based syllable tagging, rule-based word conversion, and memory-based PoS tagging. Training data from the ”Tibetan in Digital Communication” project at SOAS includes 377,000 tagged syllables and over 318,000 words with 79 PoS tags. The results show an overall accuracy of 93.0% for word segmentation with a small tag set and 92.7% with a bigger tag set, while PoS tagging attained an accuracy of 95.0%. This strategy not only effectively tackles NLP issues, but it also aids in the creation of bigger annotated corpora for Tibetan, contributing to the growth of NLP approaches in Classical Tibetan.

#### 5.8.2 Hybrid approaches

The paper Li et al. (2022) focuses on Tibetan NLP, particularly word segmentation and PoS tagging. The authors designed a unique neural network model, combining BiLSTM layers, Iterated Dilated Convolutional Neural Network (IDCNN) layers, and CRF for effective learning. Using the BIS tagging method on 500,000 records and 91 PoS Tags and a corpus of 3.6 million annotated words, the model performed in joint tagging, with an F1-score of 92.31% for word segmentation and 80.31% for PoS tagging. The paper Wang et al. (2019) presents a Tibetan PoS tagging method based on a BiLSTM CRF model. The authors created a Tibetan PoS tagset with 21 classes and 35 sub classes and trained the model on 26,228 phrases and 353,932 words, using CBOW (Continuous Bag-of-Words) generated 156,783 word vectors. The model achieved remarkable accuracy of 92.7%, demonstrating its potential for use in machine translation, speech recognition, and information retrieval for Tibetan language processing. The paper Xiangxiu et al. (2022) discussed issues in Tibetan PoS tagging, such as ambiguity, polysemy, and unregistered words, while also emphasizing the limitations of RNNs for large texts. Addressing these challenges, the authors suggest a hybrid model, ELMo + Transformer Encoder. ELMo records word features bidirectionally, which improves contextual and semantic understanding. By combining ELMo embeddings with Transformers' mechanism, the model effectively extracts semantic information and improves tagging accuracy. The dataset was collected from websites by scraping legal and educational content. Over 30,000 sentences with unique Tibetan grammar characteristics were selected. To enhance linguistic analysis, word segmentation was subsequently done on these texts using the tool, an efficient alternative (Li et al., 2015). The study used over 20,000 sentences for training and over 10,000 for testing, resulting in an impressive accuracy of around 97%.

#### 5.8.3 Result analysis

The surveyed research shows a clear development of Tibetan PoS tagging from traditional ML to sophisticated neural and hybrid architectures. The memory-based tagging framework demonstrated that the linguistic and contextual information can be successfully leveraged in both word segmentation and PoS tagging, giving a solid foundation for Tibetan NLP. The following studies employed hybrid deep learning architectures, incorporating BiLSTM, IDCNN, CRF, and transformer-based models to improve the acquisition of contextual, sequential, and semantic information. The arrival of pre-trained contextual embeddings, such as ELMo, has improved the models' capacity to deal with linguistic problems such as ambiguity, polysemy and out-of-vocabulary words. The results indicate that hybrid architectures achieve better performance than conventional machine learning approaches for modeling the complex morphology and syntax features of Tibetan. However, variations in corpus size, tagset design, and evaluation settings make direct comparison of research problematic. Overall, the shift from memory-based approaches to transformer-based designs signals the growing maturity of research in Tibetan PoS tagging and underlines the significance of contextual representations and huge annotated corpora in boosting performance. Figure 4 demonstrates the architecture of the Transformer model of PoS tagging system for the Tibetan langauge.

**Figure 4:**
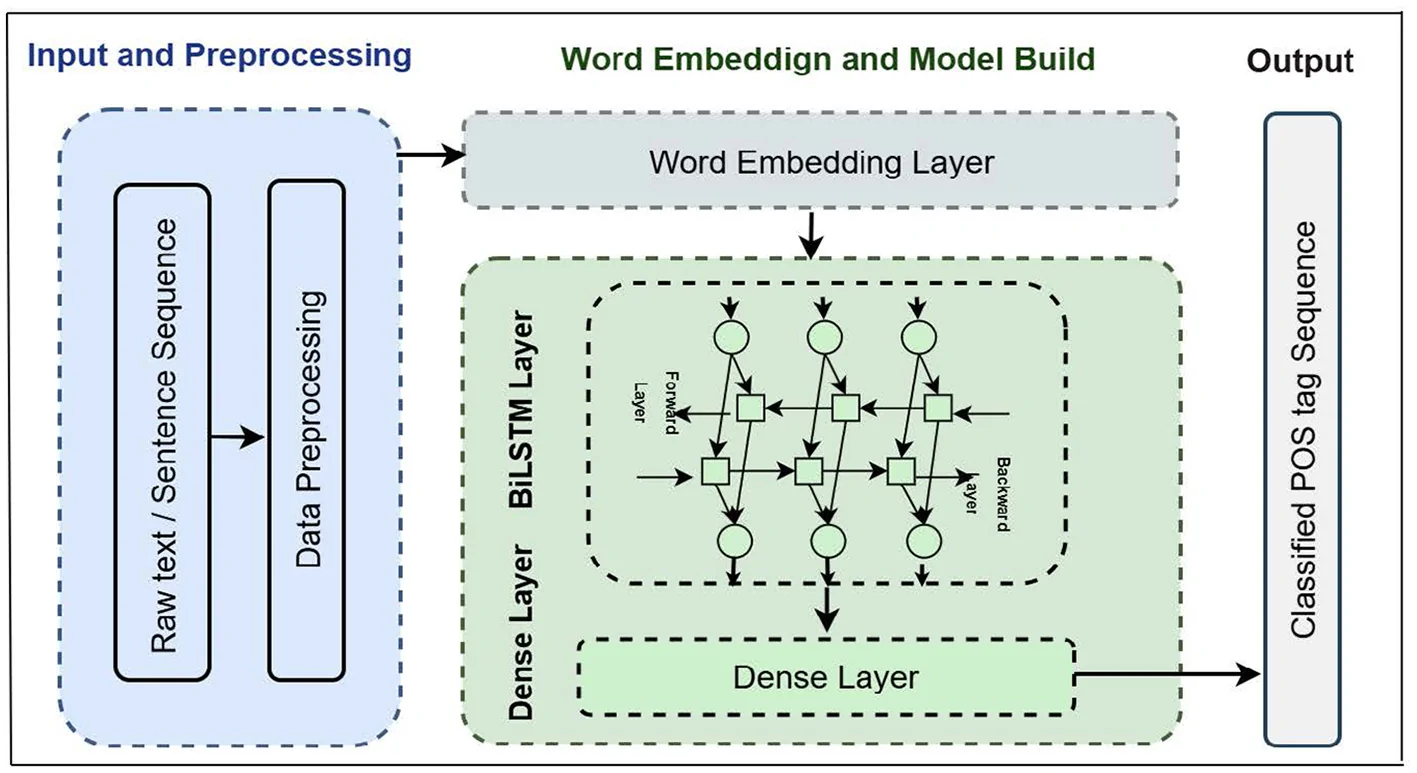
Architecture of the BiLSTM-based PoS tagger for the Bodo language, adapted from Basumatary et al. (2023).

### 5.9 Tenyidie language

#### 5.9.1 Deep learning approach

For the Tenyidie (Angami) language, a low-resource Tibeto-Burman language spoken in Nagaland, the authors Angami et al. (2025) created the first PoS tagset and PoS tagger. By presenting the first standardized PoS tagset, annotated corpus, and automated PoS tagging framework for the language, the study marks a critical turning point for Tenyidie NLP. The proposed tagset comprises 48 complete PoS tags and 22 top-level tags, specifically crafted to encapsulate the morpho-syntactic features of the extremely agglutinative Tenyidie language. According to experimental results, the CRF model performed the best, with whole and top-level tagset accuracies of 95.41% and 95.65%, respectively. By contrast, the BERT-BASE model obtained 90.78% and 92.78% for the same tagsets, while the BiLSTM model with FastText embeddings earned 93.01% and 94.07%. These findings show that for Tenyidie PoS tagging, the CRF-based method performed better than both transformer-based and DL models. CRF's better results imply that feature-based sequence labeling techniques are still quite successful for morphologically rich and low-resource languages. Even with a corpus of 255,600 tokens and 11,540 annotated phrases, deep neural architectures might not have enough training data to properly utilize contextual representations. The BiLSTM model's relatively superior performance with FastText embeddings emphasizes how crucial subword information is for managing Tenyidie's rich morphology and agglutinative nature. Even though BERT-based models showed competitive results, their decreased accuracy when compared to CRF suggests that transformer architectures could need much bigger pre-training corpora or cross-lingual transfer learning strategies to perform at their best in low-resource environments. Overall, the results show that while DL and transformer-based approaches offer promising directions for future research as larger annotated datasets and language resources become available, traditional statistical sequence-labeling approaches continue to provide strong performance for Tenyidie PoS tagging.

## 6 Dataset analysis

The performance of PoS tagging systems is strongly influenced by the quality and characteristics of the datasets used for training and evaluation. In the context of Sino-Tibetan languages, dataset construction remains a major challenge due to limited digital resources, linguistic diversity, and the scarcity of standardized annotation guidelines. This section provides an overview of datasets used in PoS tagging studies for Sino-Tibetan languages, focusing on their sources, size, annotation strategies, and tagset design. Most datasets for Sino-Tibetan PoS tagging are derived from a variety of sources, including literary texts, news articles, folklore narratives, religious texts, and manually curated corpora. In several cases, datasets have been developed through institutional or individual research efforts rather than large-scale shared tasks. As a result, publicly available and standardized corpora remain limited for many languages in this family. The size of available PoS tagged corpora for Sino-Tibetan languages varies significantly, ranging from a few thousand tokens to moderately sized datasets containing ten thousands of tokens. Due to data scarcity, many studies employ simple train-test splits, while others adopt k-fold cross-validation to maximize data utilization. The lack of uniform dataset size often makes the comparison analysis of the model performance difficult. The choice of tagset affects both accuracy and linguistic detail coarse-grained tagsets improve accuracy and comparability, while fine-grained tagsets capture richer grammatical features but are harder to annotate, especially for Sino-Tibetan languages with particles and classifiers. Dataset creation for Sino-Tibetan languages faces several challenges, including limited annotated data, inconsistencies in annotation schemes, dialectal variation, and the absence of shared benchmarks. These issues highlight the need for standardized datasets and annotation guidelines to support reproducibility and fair comparison of PoS tagging approaches. Tables 1, 2 provides a comparative summary of the datasets used in the reviewed studies. The table highlights the language, dataset source, corpus size, tagset, approach, and state of art performance. The size and annotation procedures of the reviewed corpora differ significantly. Dataset sizes range from a few thousand tokens to several thousands of tokens, reflecting the low-resource nature of many Sino-Tibetan languages. The investigation finds large disparities in tagset design between languages. While some research embrace coarse-grained tagsets to increase annotation consistency and tagging accuracy, others employ fine-grained tagsets to capture more linguistic information. However, larger tagsets often increase annotation complexity and make the PoS tagging operation more burdensome. Furthermore, differences in annotation schemes and evaluation protocols hinder direct comparison of reported results among investigations. Dataset size also effects the model performance. Research utilizing larger and more diverse corpora generally report more robust and stable results, whereas studies based on smaller datasets often face challenges related to data sparsity and limited generalization capability. Consequently, performance discrepancies should be viewed not only in terms of the tagging process but also with respect to the underlying dataset features. Several problems remain evident across the analyzed datasets, including inadequate annotated resources, dialectal variety, script diversity, inconsistent annotation guidelines, and the absence of shared benchmark datasets. These issues hinder reproducibility and make meaningful comparison across PoS tagging systems impossible. Therefore, the establishment of standardized corpora, uniform annotation systems, and publicly accessible benchmark datasets remains a significant need for the future research for NLP task like PoS tagging in Sino-Tibetan languages.

**Table 1:** Overview of datasets (I).

Papers	Languages	Dataset sources	Dataset size	Tagset	Performnace
Minn and Soe (2019)	Myanmar	Wikipedia, MCF dataset, Myanmar Grammar Book	300 sentences	14 tags	Accuracy:80.00
Myint (2011)	Myanmar	UCSM	400 sentences	88 tags	Accuracy:84.71%
Lam and Soe (2020)	Myanmar	Online journals, newspapers, e-books	51,207 sentences	12 tags	Accuracy:94.00%
Linn and Patil (2019)	Kayah	Manually annotated with translator support	3,994 words	16 tags	Accuracy:87.00%
Singha et al. (2012a)	Manipuri	Rule-based handwritten corpus	1,000 words	97 tags	Accuracy:85.00%
Singh (2008)	Manipuri	Manually annotated	63,200 tokens	26 tags	Accuracy:74.38%
Singh and Bandyopadhyay (2008)	Manipuri	Dictionaries (root, prefix, suffix, bilingual)	3,784 tokens	13 tags	Accuracy:69.00%
Nongmeikapam et al. (2010)	Manipuri	Web newspapers, manually tagged	40,000 words	Not Reported(NR)	F1-score:73.68%
Singha et al. (2012b)	Manipuri	Rule-based PoS tagger	2,000 words	97 tags	Accuracy:80.00%
Pakray et al. (2015)	Mizo	Mizo to English dictionary	26,407 words	24 PoS	–
Nunsanga et al. (2021)	Mizo	Vanglaini newspaper corpus	30,000 words	47 tags	F1-score:89.30%
Nunsanga et al. (2023)	Mizo	Collected from diverse web sources and Mannually annotated the text	72,077 tokens	48 tags	F1-score: 79.64%
Patra et al. (2012)	Kokborok	Manually created	42,537 words	26 tags	Accuracy:70.00%

**Table 2:** Overview of datasets (II).

Papers	Languages	Dataset sources	Dataset size	Tagset	Performnace
Liao et al. (2015)	Classical Chinese	Manually created	4,240 words	NR	F1-score:86.20%
Shao et al. (2017)	Classical Chinese	Penn Treebank, UD Chinese corpus	99k–1.7M	33, 17 tags	F1-score:94.38%
Shen et al. (2022)	Ancient Chinese	Ancient Chinese text/Siku Quanshu	NR	22 tags	F1-score:88.12%(in domain), 78.31%(out domain)
Feng and Li (2023)	Ancient Chinese	Zuozhuan	8,701 sentences	22 tags	F1-score:90.55%(TestA), 86.21%(TestB)
Xu and Wang (2022)	Chinese	MSR, CTB7, CTB5	494K, 78K, 2131K	NR	Micro F1:88.4%(MSR)
Siram et al. (2022a)	Nyishi	Nyishi-English dictionary	25,000 words	36 tags	–
Siram et al. (2022b)	Nyishi	Manually created	30,000 characters	36 tags	Accuracy:89.00%
Basumatary et al. (2023)	Bodo	NPLT	9,991 sentences	36 tags	Accuracy:97.05
Pathak et al. (2025)	Bodo	ILCI Phase-II	24,003 sentences	34 tags	F1-score:80.41%
Li et al. (2022)	Tibetan	Northwest Minzu University corpus	3.6M words	91 tags	F1-score:81.26%
Meelen and Hill (2017)	Tibetan	Tibetan in Digital Communication project	318,000 words	NR	Accuracy:95.00%
Wang et al. (2019)	Tibetan	News, broadcasting, articles (manually tagged)	26,228 sentences	35 tags	Accuracy:92.70%
Xiangxiu et al. (2022)	Tibetan	Mixed sources, manually tagged	20,000 sentences	NR	Accuracy:97.00%
Angami et al. (2025)	Tenyidie (Angami)	News articles and literature books	11,540 sentences	48 tags	Accuracy:95.41%

## 7 Performance evaluation metrics for PoS taggers

Evaluation metrics are important for measuring the performance of PoS tagging systems and for comparing different approaches in an objective manner. In works like tagging on Sino-Tibetan languages, traditional ML and DL algorithms are often evaluated with standard classification metrics such as accuracy, precision, recall and F1-score. Accuracy gives an overall measure of the accurate tag assign to it, while precision and recall provide a more thorough evaluation of the model's ability to correctly identify the individual PoS categories. The F1-score, which balances precision and recall, is especially effective when the distribution of PoS tags is imbalanced. Such criteria are even more relevant in the case of Sino-Tibetan languages, many of which are low-resource and have extremely imbalanced tag distributions. In this context, mere dependence on accuracy may be deceptive, as strong performance on commonly occurring tags may disguise poor performance on less frequent yet linguistically pertinent categories. Therefore, precision, recall and F1-score give a more complete evaluation of the model behavior across the multiple PoS classes. Moreover, other research use confusion matrices and tag-wise performance analysis to pinpoint particular causes of tagging errors and to better understand the strengths and limitations of different models. Standardized evaluation criteria are necessary to benchmark PoS tagging systems on different Sino-Tibetan languages and experimental scenarios. Existing studies generally employ multiple corpora, tagsets and data sizes. Consistent reporting of evaluation measures promotes a fairer comparison of methodologies and helps find the most effective approaches. Moreover, thorough evaluation assists in the creation of more robust tagging systems by highlighting the areas where further improvements in data resources, feature representation or model design are needed.

### 7.1 Accuracy

Accuracy is a commonly used evaluation metric in PoS tagging that quantifies the ratio of correctly labeled tokens; however, it may inadequately represent performance on infrequent PoS categories, particularly in low-resource Sino-Tibetan languages (Equation 1).


$$\begin{array}{l} \text{Accuracy}=\frac{\text{Number of correctly predicted instances}}{\text{Total number of instances}}\times 100 \end{array}$$ (1)

### 7.2 Precision

Precision evaluates the accuracy of predicted PoS tags in a particular category (Equation 2).


$$\begin{array}{l} \text{Precision}=\frac{\text{TP}}{\text{TP + FP}} \end{array}$$ (2)

Where TP denotes True Positives and FP denotes False Positives.

### 7.3 Recall

Recall quantifies the tagger's ability to accurately identify all instances of a particular PoS category (Equation 3).


$$\begin{array}{l} \text{Recall}=\frac{\text{TP}}{\text{TP + FN}} \end{array}$$ (3)

where, FN denotes False Negatives.

### 7.4 F1_Score

The F1-score represents the harmonic mean of precision and recall, serving as an equitable evaluation measure (Equation 4).


$$\begin{array}{l} F_{1}=2\times \frac{\text{Precision }\times \text{ Recall}}{\text{Precision }+\text{ Recall}} \end{array}$$ (4)

F1-scores are often provided as macro-averaged or micro-averaged values in PoS tagging assignments to take tag imbalance into consideration.

## 8 Comparative analysis of PoS tagging approaches for Sino-Tibetan languages

Table 3 summarizes the main PoS tagging methods for Sino-Tibetan languages, including their core techniques, data needs, strengths, limits, and applicability for low-resource language processing in a comparative way. To systematically classify the PoS tagging approaches explored in this study, Figure 2 presents a proposed taxonomy of PoS tagging approaches for Sino-Tibetan languages. The taxonomy classifies the reviewed approaches into rule-based, statistical, machine learning, deep learning, transformer-based, and hybrid approaches, showcasing the methodological advancement of PoS tagging research from conventional language practices to modern neural architectures. This categorization acts as a conceptual foundation for the comparative discussion provided in this section. Rule-based approaches demonstrate the initial PoS tagging techniques for Sino-Tibetan languages. Such systems depend on constructed linguistic rules, morphological analyzers and expert knowledge, and are hence suited for languages with scarce annotated corpora. Rule-based approaches require little training data but is a tedious job to create and often have difficulty with linguistic ambiguity, unseen terms and domain adaptation. Statistical approaches, such as HMM, N-gram frameworks, and Markov Logic Networks, represented a notable progression by automatically learning contextual relationships from labeled datasets. These models reduce manual feature construction and are more scalable than rule-based systems. But their efficacy is primarily dependent on availability of annotated training data and the ability to model limited contextual information, making them less effective for complicated language structures. The application of ML algorithms, especially the SVM and CRF, Memory-based tagging approaches, considerably enhanced the performance of PoS tagging by combining the lexical, contextual and morphological data. They are more flexible than strictly statistical models, but still require careful feature selection and linguistic skill. Feature sparsity and domain-specific variances may also affect their performance. DL approaches such as RNN, GRU LSTM and BiLSTM architectures automatically learn contextual and sequential feature representations from annotated data, thereby overcoming several limitations of classic machine learning algorithms, particularly their reliance on manual feature engineering. Their capacity to identify sequential dependencies and contextual features has markedly enhanced PoS tagging efficacy for several Sino-Tibetan languages. However, these models typically require sufficiently extensive annotated datasets to achieve robust generalization, which remains a major challenge for many low-resource languages. Recent research has initiated explorations into transformer-based approaches for PoS tagging in specific Sino-Tibetan languages. Transformer-based architectures, such as BodoBERT, SikuRoberta, and those utilizing Transformer Encoders, utilize contextualized representations and self-attention processes to more efficiently capture long-range semantic and grammatical relationships compared to recurrent neural networks. As a result, these models typically demonstrate enhanced contextual comprehension and better generalization. However, their efficacy is depends on availability of high-quality pre-trained resources, considerable computing resources, and sufficiently extensive annotated datasets, which are still scarce for numerous under-resourced Sino-Tibetan languages. Hybrid approaches combine the strengths of multiple methodologies to address the limitations of individual techniques. The reviewed studies employ architectures such as Rule-based + CRF, HMM + N-gram + Rule-based, BiLSTM + IDCNN + CRF, BiLSTM-CRF + CBOW, ELMo + Transformer, BodoBERT + BPE + BiLSTM-CRF, and sikuRoberta + CRF. By integrating complementary approaches for contextual representation learning and structured sequence labeling in hybrid models often results in enhanced robustness and improved handling of language ambiguity, morphological complexity, and contextual interdependence. Overall, the reviewed research show that hybrid architectures are among the most promising options for enhancing PoS tagging performance in low-resource Sino-Tibetan languages. The studied research indicates that enhancements in PoS tagging performance are not only influenced by the selection of model design. Model effectiveness is significantly affected by different factors such as corpus size, annotation quality, annotation consistency, tagset complexity, and the linguistic features of particular Sino-Tibetan languages. Traditional rule-based and statistical methodologies continue to be advantageous for languages with limited annotated resources, as they require less training data and computational resources. Machine learning techniques enhance tagging quality through discriminative learning but depend on manually created features. Deep learning frameworks improve efficacy by automatically learning contextual and sequential representations from data, whereas transformer-based and hybrid models provide enhanced contextual comprehension through self-attention mechanisms and the integration of complementary methodologies. However, the enhancements in performance achieved by these sophisticated designs are significantly depends on the presence of high-quality annotated datasets and sufficient computational resources. The reviewed literature indicates that effective PoS tagging for Sino-Tibetan languages relies not solely on the choice of a suitable model architecture but also on enhancing corpus quality, annotation criteria, and resource accessibility. To demonstrate the methodological progression of PoS tagging systems for Sino-Tibetan languages, representative architectures from different research approaches are presented in Figures 3–5. Figure 3 illustrates the SVM-based architecture for Kokborok from Patra et al. (2012), representing traditional machine learning methodologies that depend on manually designed contextual and morphological features. Figure 4 depicts the BiLSTM architecture designed for Bodo (Basumatary et al., 2023), emphasizing a transition to deep learning techniques that automatically acquire contextual representations from data. Figure 5 illustrates a transformer-based architecture for Tibetan (Xiangxiu et al., 2022), showcasing recent progress in contextualized representation learning and self-attention processes. These architectures were chosen since they illustrate the evolution from traditional machine learning techniques to deep learning and transformer-based methods in Sino-Tibetan PoS tagging research.

**Table 3:** Comparison of PoS tagging approaches for Sino-Tibetan languages.

Approaches	Models	Data requirement	Advantages	Limitations	Suitability
Rule-based	Manually defined linguistic rules	No annotated data	Interpretable; high precision	Poor scalability; low coverage	Suitable for very low-resource languages and initial system development
Statistical	HMM, N-gram, Markov logic Networks	Small annotated corpora	Simple probabilistic modeling	Limited contextual modeling	Baseline systems
Machine learning	CRF, SVM, Memory-based tagger	Medium annotated corpora	Effective feature-based learning	Manual feature engineering	Moderate-resource settings
Deep learning	RNN, GRU, LSTM, BiLSTM	Medium to large corpora	Automatic feature learning,captures contextual and sequential dependencies	Data-hungry; Computationally expensive	High performance with sufficient data
Hybrid	BiRNN-CRF ELMo + Transformer, BodoBERT + BPE + BiLSTM-CRF	Medium to large annotated corpora	Improved robustness and contextual modeling	Complex design	Morphologically rich low-resource languages
Transformer based	BodoBERT, sikuRoberta, Transformer Encoder	Pre-trained language model + annotated fine-tuning corpus	Contextual representations, long-range dependency modeling	High computation cost, limited pre-trained resources	Promising for low-resource transfer learning

**Figure 5:**
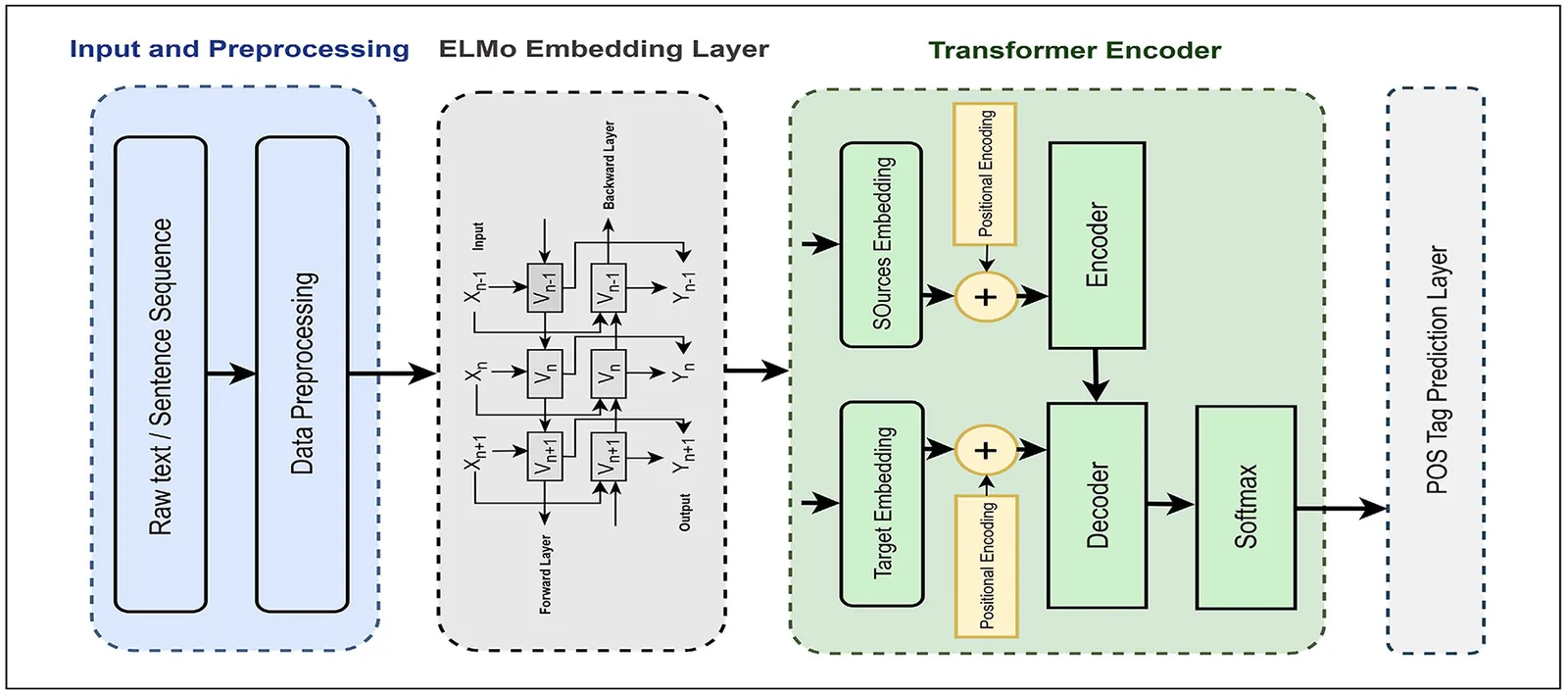
Architecture of transformer model of PoS tagging system for the Tibetan language, adapted from Xiangxiu et al. (2022).

## 9 Analysis

The literature reviewed demonstrates a clear evolution in the PoS tagging approaches for the Sino-Tibetan languages, from rule-based and statistical methods to ML, DL, transformer-based and hybrid architectures. The early approaches relied on handcrafted linguistic rules and statistical sequence models that performed well on relatively small annotated datasets but required a lot of feature engineering and linguistic expertise. Conversely, recent techniques are more and more based on contextualized representations and neural sequence-labeling frameworks which learn linguistic patterns automatically from annotated data. The comparative results illustrated in Tables 4, 5, along with the performance analysis shown in Figure 6, demonstrate significant differences in PoS tagging performance among various Sino-Tibetan languages, methodologies, datasets, and evaluation criteria. This variation indicates that the performance of PoS tagging is affected by both the foundational model architecture and elements like corpus size, annotation quality, tagset complexity, and language-specific features. Traditional methodologies, such as HMM, CRF, and SVM, frequently attain promising results on modestly sized annotated datasets by employing meticulously created language attributes and statistical sequence analysis. As a result, these methodologies continue to be practical choices for languages that possess scarce annotated resources. On the other hand, DL and transformer-based models usually perform better with large annotated datasets, as they can automatically learn richer contextual and semantic representations. These models are able to better capture long-range contextual dependencies compared to traditional approaches, resulting in improved PoS tagging performance for different Sino-Tibetan languages. However, the reviewed studies show that larger datasets alone do not necessarily lead to better PoS tagging outcomes. Some studies observed only moderate effectiveness even with reasonably large corpora because of complicated tagsets, linguistic ambiguities, class imbalance, inconsistent annotation, and domain-specific problems. In contrast, other works with smaller but well-annotated datasets showed good performance. This findings demonstrates that corpus quality, annotation consistency, and tagset design as criteria that are equivalent to the selection of a PoS tagging approaches. Moreover, the comparative evaluation suggests that enhancements in PoS tagging performance cannot be exclusively attributed to increased model complexity. DL models improve feature representation by the automatic acquisition of contextual and sequential data, whereas transformer designs enhance contextual comprehension through the utilization of self-attention processes and contextualized representations. Hybrid methodologies utilize the complementary strengths of different techniques, including contextual representation learning and CRF-based sequence optimization, to enhance sequence-labeling performance. Consequently, the effectiveness of PoS tagging systems depends on the combination of model architecture, corpus quality, annotation consistency, and the accessibility of meticulously annotated linguistic resources, rather than on any individual element. One of the main challenges found across the examined studies is the lack of consistent benchmark datasets and uniform evaluation methodologies for Sino-Tibetan languages. Most of the studies use language-specific corpora, modified tagsets and varied evaluation procedures which make the accuracies and F1-scores impossible to compare directly. Hence, a better performance reported in one study does not mean that a model is more successful than another employing a different corpus, annotation scheme or evaluation scenario. Therefore, reported findings should be taken carefully observe in terms of methodology and dataset discrepancies. To summarize, the literature review,it shows a trend toward contextualized representation learning, transformer-based models and hybrid architectures for Sino-Tibetan language PoS tagging. These approaches have shown great promise in enhancing the accuracy and robustness of tagging especially in morphologically rich and low resource languages. However, their performance still relies heavily on the availability of high quality language resources, which emphasizes the need for corpus development, benchmark datasets and consistent evaluation techniques for future research. Table 6 shows that the studies published from 2021 to 2025 mostly utilize deep learning, transformer-based and hybrid techniques, representing the current research trends in Sino-Tibetan language PoS tagging. Furthermore the highest performance reported by several PoS tagging approaches for Sino-Tibetan languages is presented in Table 7, offering a comprehensive overview of the efficacy of different techniques.

**Table 4:** Performance of different PoS taggers for Sino-Tibetan languages (I).

Languages	Papers	Approaches/tools	Corpus/dataset	Performance(%)
Myanmar	Minn and Soe, 2019	N-gram, rule-based	300 sentences	80.00
Myanmar	Myint, 2011	Rule-based + probabilistic + CRF	400 sentences	84.71
Myanmar	Lam and Soe, 2020	HMM	51,207 sentences	94.00
Kayah	Linn and Patil, 2019	Viterbi algorithm + HMM	3,994 words	87.00
Manipuri	Singha et al., 2012a	Rule-based	1,000 words	85.00
Manipuri	Singh, 2008	CRF, SVM	63,200 tokens	72.04, 74.38
Manipuri	Singh and Bandyopadhyay, 2008	Morphology-driven	3,784 tokens	69.00
Manipuri	Nongmeikapam et al., 2010	CRF	40,000 words	73.68(F1-score)
Manipuri	Singha et al., 2012b	HMM	2,000 words	80.00
Mizo	Nunsanga et al., 2021	CRF	30,000 words	89.30(F1-score)
Mizo	Nunsanga et al., 2023	HMM+N-gram+Rule-based	72,077 words	79.64(F1-score)
Kokborok	Patra et al., 2012	Rule-based	42,537 words	70.00

**Table 5:** Performance of different PoS taggers for Sino-Tibetan languages (II).

Languages	Papers	Approaches/tools	Corpus/dataset	Performance(%)
Classical Chinese	Liao et al., 2015	Markov logic networks	4,240 words	86.20 (F1-score)
Classical Chinese	Shao et al., 2017	BiRNN-CRF	99k–1.7M	94.38 (F1-score)
Ancient Chinese	Shen et al., 2022	SikuRoberta + CRF with LSTM and MLM-based augmentation	Note Reported	88.12(F1-score)
Ancient Chinese	Feng and Li, 2023	SikuRoberta + CRF with Distant Supervision and Relabeling	8,701 sentences	90.55(F1-score)
Chinese	Xu and Wang, 2022	Word2Vec+Attention-based AlexNet + Neural Language Model + CRF	NR	88.4(Micro-F1)
Nyishi	Siram et al., 2022b	HMM	30,000 characters	89.00
Bodo	Basumatary et al., 2023	RNN, GRU, LSTM, BiLSTM	9,991 sentences	92.37, 92.22, 92.91, 97.05
Bodo	Pathak et al., 2025	BodoBERT + BPE + BiLSTM-CRF	24,003 sentences	80.41 (F1-score)
Tibetan	Li et al., 2022	BiLSTM + IDCNN + CRF	3.6M words	81.26 (F1-score)
Tibetan	Meelen and Hill, 2017	Memory-based	318,000 words	95.00
Tibetan	Wang et al., 2019	BiLSTM-CRF + CBOW	26,228 sentences	92.70
Tibetan	Xiangxiu et al., 2022	ELMo + Transformer	20,000 sentences	97.00
Tenyidie (Angami)	Angami et al., 2025	CRF	11,540 sentences	95.41

**Figure 6:**
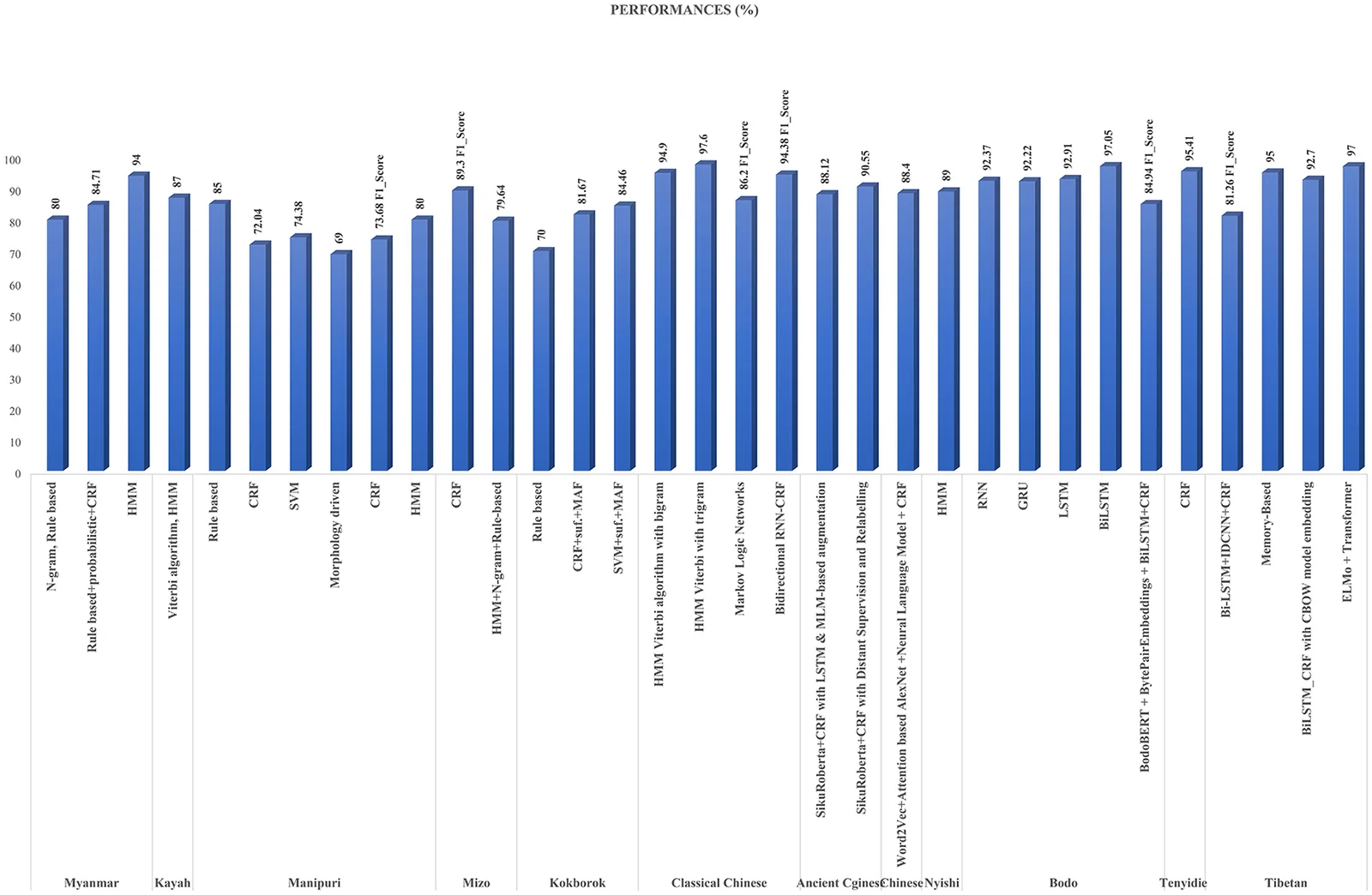
Bar siagram of the performance of different PoS taggers for Sino-Tibetan languages.

**Table 6:** Recent works on selected languages (2021–2025).

Authors	Languages	Approaches/models	Datasets/tagset	Performance
Nunsanga et al. (2021)	Mizo	CRF	40,000 words, 41 tags	F1-score: 73.68%
Siram et al. (2022b)	Nyishi	HMM	30,000 characters, 36 tags	Accuracy: 89%
Li et al. (2022)	Tibetan	BiLSTM + IDCNN + CRF	3.6M words, 91 tags	F1-score: 81.26%
Xiangxiu et al. (2022)	Tibetan	ELMo + Transformer	30,000 sentences	Accuracy: 97%
Feng and Li (2023)	Ancient Chinese	SikuRoberta + CRF with Distant Supervision and Relabeling	8,701 sentences	F1-score: 90.55%
Basumatary et al. (2023)	Bodo	RNN, GRU, LSTM, BiLSTM	9,991 sentences, 36 tags	Accuracy: 92.37%, 92.91%, 92.22%, 97.05%
Pathak et al. (2025)	Bodo	BERT + Byte-Pair Encoding + BiLSTM-CRF	24,003 sentences, 34 tags	F1-score: 84.94%
Angami et al. (2025)	Tenyidie	CRF	11,540 sentences	Accuracy: 95.41%

**Table 7:** Highest performance achieved by different approaches for the Sino-Tibetan languages.

Approaches	Language (references)	Performance
Rule-based	Manipuri (Singha et al., 2012a)	Accuracy: 85%
Statistical	Manipuri (Lam and Soe, 2020)	Accuracy: 94%
Machine learning	Tibetan (Angami et al., 2025)	Accuracy: 95.41%
Deep learning	Bodo (Basumatary et al., 2023)	Accuracy: 97.05%
Hybrid	Tibetan (Xiangxiu et al., 2022)	Accuracy: 97%

### 9.1 Limitation of the review

The primary objective of this review is to provide a comprehensive overview of research on PoS tagging for Sino-Tibetan languages. However, several limitations were acknowledged when interpreting the findings. A major challenge encountered during this review was the limited availability of peer-reviewed journal articles on PoS tagging for many Sino-Tibetan and other low-resource languages. Research in this domain is still emerging, and a substantial proportion of the available literature is considered from conference and workshop papers. While every effort was made to prioritize peer-reviewed journal articles, the scarcity of such publications for several languages constrained the extent of comparative analysis and synthesis. Another limitation arises from the considerable variation across the reviewed studies. The existing research differs significantly in terms of corpus size, annotation guidelines, tagset design, feature representation, model architecture, and evaluation methodology. Furthermore, the lack of standardized benchmark datasets for most Sino-Tibetan languages makes it difficult to perform direct and fair comparisons among different PoS tagging approaches. The reviewed studies also report performance using different evaluation metrics, such as accuracy, precision, recall, and F1-score, making quantitative comparison across studies less straightforward. In addition, variations in linguistic characteristics, annotation standards, and tagset definitions across Sino-Tibetan languages further complicate the interpretation and comparison of reported results Despite these limitations, this review provides a systematic synthesis of the existing literature on PoS tagging for Sino-Tibetan languages. It summarizes the major methodological developments, identifies current research trends, highlights the key challenges faced by researchers, and discusses important research gaps. The findings presented in this review are expected to serve as a useful reference for future studies and to encourage the development of more robust and effective PoS tagging techniques for low-resource Sino-Tibetan languages.

### 9.2 Research gap and future directions

The studied literature shows many gaps in research on PoS tagging for the Sino-Tibetan language family. Recent improvements in ML, DL and transformer-based techniques have not been able to address the problem that most of the Sino-Tibetan languages are under-resourced due to the lack of high-quality annotated corpora. Furthermore, for many languages defined benchmark datasets, unified annotation criteria and consistent tagsets are still missing, which makes fair comparison, repeatability and benchmarking of diverse PoS tagging systems difficult. Several Sino-Tibetan languages have shown promising performance with DL, transformer-based, and hybrid approaches, although they are still only applied to a few languages, while many languages have only been studied with rule-based or statistical methods. Moreover, the literature study indicates a lack of research on multilingual pre-trained language models, transfer learning, cross-lingual learning and few-shot learning techniques which can be very useful for low-resource PoS tagging. Similarly, the use of contemporary large language models (LLMs) and prompt-based learning for PoS tagging in Sino-Tibetan languages is also relatively unexplored and is an attractive avenue for future research. Another significant difficulty is the creation of robust PoS tagging systems capable of managing noisy corpora, unseen or out-of-vocabulary words, inconsistent or incomplete annotations, domain-specific text, and informal language used in social media. Addressing these practical issues requires cleaner benchmark corpora, uniform annotation techniques, domain-diverse datasets, and robust sequence-labeling algorithms that generalize well across multiple text domains. Therefore, future research should take into account the construction of high-quality annotated corpora, the development of benchmark datasets and standard evaluation protocols, as well as comprehensive comparisons of machine learning, deep learning, transformer-based and hybrid approaches under a consistent experimental setting. A comprehensive evaluation of these approaches on Bodo and other low-resource Sino-Tibetan languages would be very informative regarding their effectiveness in low-resource settings in particular. To summarize, this review covers the available literature, points out gaps in current research, suggests possible methodological approaches, and provides a basis for future research on robust PoS tagging systems for Sino-Tibetan languages.

## 10 Conclusion

Natural Language Processing has shown substantial progress in recent decades, resulting in the creation of many linguistic resources and applications for multiple global languages. Nevertheless, numerous Sino-Tibetan languages persist in being under-resourced owing to the lack of digital content, annotated corpora, and standardized linguistic resources. This review thoroughly analyzed the available research on PoS tagging for Sino-Tibetan languages, including Myanmar, Manipuri, Mizo, Kokborok, Chinese, Nyishi, Bodo, Tibetan, and Tenyidie, as PoS tagging is a crucial component in numerous NLP applications. The reviewed studies demonstrate a distinct evolution from rule-based and statistical methodologies to machine learning, deep learning, transformer-based models, and hybrid architectures. Although conventional methods are beneficial in resource-limited settings, contemporary research increasingly utilizes contextualized embeddings, transformer-based architectures, and hybrid sequence-labeling frameworks to enhance tagging efficacy. The comparison reveals that the efficacy of PoS tagging is affected by model architecture, corpus size, annotation quality, tagset design, and linguistic complexity. The paper emphasizes on increasing significance of pre-trained language models and transfer-learning methodologies for mitigating data scarcity in low-resource languages. Despite recent advancements, limitations still exist, including the absence of extensive annotated corpora, defined benchmark datasets, uniform annotation methodologies, and language-specific pre-trained resources. These constraints impede direct comparisons among research and hamper the advancement of strong and generalizable PoS tagging systems. Thus, subsequent research must to concentrate on corpus building, benchmark establishment, multilingual and cross-lingual learning, as well as the adaption of sophisticated transformer-based architectures for low-resource Sino-Tibetan languages. This study presents a thorough examination of PoS tagging research for Sino-Tibetan languages, highlights prevailing trends and constraints, and gives significant insights for academics engaged in low-resource language processing. The results of this evaluation can provide the groundwork for future research focused on creating more precise and robust PoS tagging systems for underrepresented Sino-Tibetan languages.

## References

[B1] Ahmed M. Talukdar K. Boruah P. Sarma S. K. Kashyap K. (2023). “Guit-nlp's submission to shared task: low resource indic language translation,” in Proceedings of the eighth conference on machine translation, (Singapore: ACL-WMT). 935–940. doi: 10.18653/v1/2023.wmt-1.87

[B2] Ahmed M. A. Kashyap K. Sarma S. K. (2023). “Pre-processing and resource modelling for english-assamese nmt system,” in 2023 4th international conference on computing and communication systems (I3CS) (Shillong: IEEE), 1–6. doi: 10.1109/I3CS58314.2023.10127567

[B3] Angami T. Ezung M. K. Singh S. R. Tuithung T. Singh L. G. (2025). Development of part-of-speech tagset and tagger for the tenyidie (angami) language. Res. Square. doi: 10.21203/rs.3.rs-7613811/v1

[B4] Barman A. K. Sarmah J. Sarma S. K. (2013). “Wordnet based information retrieval system for assamese,” in 2013 UKSim 15th international conference on computer modelling and simulation (London: IEEE), 480–484. doi: 10.1109/UKSim.2013.90

[B5] Baruah K. K. Das P. Hannan A. Sarma S. K. (2014). Assamese-english bilingual machine translation. arXiv Preprint arXiv:1407.2019. doi: 10.48550/arXiv.1407.2019

[B6] Baruah N. Gogoi A. Neog M. (2023a). “Detection of hate speech in assamese text,” in International conference on communication and computational technologies (Jaipur: Springer), 655–670. doi: 10.1007/978-981-99-3485-0_52

[B7] Baruah N. Gogoi A. Phukan R. Goutom P. J. (2023b). “Hate speech detection for a low level language (Assamese),” in 2023 14th international conference on computing communication and networking technologies (ICCCNT) (Delhi: IEEE), 1–6. doi: 10.1109/ICCCNT56998.2023.10307931

[B8] Baruah N. Gogoi A. Sarma S. K. Borah R. (2021a). “Utilizing corpus statistics for assamese word sense disambiguation,” in Advances in computing and network communications, eds. S. M. Thampi, E. Gelenbe, M. Atiquzzaman, V. Chaudhary, and K.-C. Li (Singapore: Springer Singapore), 271–283. doi: 10.1007/978-981-33-6987-0_23

[B9] Baruah N. Sarma S. Borkotokey S. (2021b). “A Single Document Assamese Text Summarization Using a Combination of Statistical Features and Assamese WordNet,” in Progress in Advanced Computing and Intelligent Engineering. Advances in Intelligent Systems and Computing, vol. 1199, eds.C. R. Panigrahi, B. Pati, P. Mohapatra, R. Buyya, and K. C. Li, (Singapore: Springer), 125–136. doi: 10.1007/978-981-15-6353-9_12

[B10] Baruah N. Sarma S. K. Borkotokey S. (2019). “A novel approach of text summarization using assamese wordnet,” in 2019 4th international conference on information systems and computer networks (ISCON) (Mathura: IEEE), 305–310. doi: 10.1109/ISCON47742.2019.9036285

[B11] Baruah N. Sarma S. K. Borkotokey S. (2020). Evaluation of content compaction in assamese language. Proc. Comput. Sci. 171, 2275–2284. doi: 10.1016/j.procs.2020.04.246

[B12] Baruah N. Sarma S. K. Borkotokey S. Borah R. Phukan R. D. Gogoi A. (2022). “A graph-based extractive assamese text summarization,” in Computational methods and data engineering: proceedings of ICCMDE 2021 (Vellor: Springer), 1–12. doi: 10.1007/978-981-19-3015-7_1

[B13] Basumatary B. Rahman M. Sarma S. K. Boruah P. A. Talukdar K. (2023). “Deep learning based bodo parts of speech tagger,” in 2023 14th International conference on computing communication and networking technologies (ICCCNT) (IEEE), 1–5. doi: 10.1109/ICCCNT56998.2023.10308365

[B14] Brahma B. Barman A. Sarma S. K. Boro B. (2012). “Corpus building of literary lesser rich language-bodo: insights and challenges,” in Proceedings of the 10th workshop on Asian language resources (Mumbai: IIT Mumbai), 29–34.

[B15] Feng S. Li P. (2023). “Ancient chinese word segmentation and part-of-speech tagging using distant supervision,” in ICASSP 2023-2023 IEEE international conference on acoustics, speech and signal processing (ICASSP) (IEEE), 1–5. doi: 10.1109/ICASSP49357.2023.10097080

[B16] Gayary J. A. Sarma S. K. Sarma H. K. D. Talukdar K. (2025). Named entity recognition (ner) for sino-tibetan languages: a comprehensive review and status. Comput. Sist. 29, 1179–1195. doi: 10.13053/cys-29-3-5896

[B17] Gogoi A. Baruah N. (2022). A lemmatizer for low-resource languages: WSD and its role in the assamese language. Trans. Asian Low-Resour. Lang. Inform. Process. 21, 1–22. doi: 10.1145/3502157

[B18] Gogoi A. Baruah N. Nath L. J. (2021). Assamese word sense disambiguation using cuckoo search algorithm. Proc. Comput. Sci. 189, 142–147. doi: 10.1016/j.procs.2021.05.110

[B19] Gogoi A. Baruah N. Sarma S. K. (2020). “Assamese word sense disambiguation using genetic algorithm,” in Proceedings of the 17th international conference on natural language processing (ICON) (Patna: NLPAI), 303–307.

[B20] Goutom P. J. Baruah N. Sonowal P. (2023). An abstractive text summarization using deep learning in assamese. Int. J. Inform. Technol. 15, 2365–2372. doi: 10.1007/s41870-023-01279-7

[B21] Htike K. W. W. Thu Y. K. Zhang Z. Pa W. P. Sagisaka Y. Iwahashi N. (2017). “Comparison of six pos tagging methods on 10k sentences myanmar language (burmese) pos tagged corpus,” in Proceedings of the CICLING (Budapest: Springer).

[B22] Jimmy L. Nongmeikappam K. Naskar S. K. (2023). Bilstm-crf manipuri ner with character-level word representation. Arab. J. Sci. Eng. 48, 1715–1734. doi: 10.1007/s13369-022-06933-z

[B23] Kashyap K. Sarma S. K. Ahmed M. A. (2024). Improving translation between english, assamese bilingual pair with monolingual data, length penalty and model averaging. Int. J. Inform. Technol. 16, 1539–1549. doi: 10.1007/s41870-023-01714-9

[B24] Laishram J. Nongmeikapam K. Naskar S. K. (2020). “Deep neural model for manipuri multiword named entity recognition with unsupervised cluster feature,” in Proceedings of the 17th international conference on natural language processing (ICON) (Patna: NLPAI), 420–429.

[B25] Lam C. D. Soe K. M. (2020). Building Large Scale Text Corpus for Joint Word Segmentation and Part-of-Speech Tagging of Myanmar Language (PhD thesis). MERAL Portal. University of Computer Studies, Myanmar.

[B26] LaPolla R. J. Thurgood G. (2006). The Sino-Tibetan Languages, 1st edn. Routledge. doi: 10.4324/9780203221051

[B27] Li Y. Jiang J. Jia Y. Yu H. (2015). Tip-las: An open source toolkit for tibetan word segmentation and pos tagging. Comput. J. Chin. Inform. 29, 203–207.

[B28] Li Y. Li X. Wang Y. Lv H. Li F. Duo L. (2022). Character-based joint word segmentation and part-of-speech tagging for tibetan based on deep learning. Trans. Asian Low-Resour. Lang. Inform. Process. 21, 1–15. doi: 10.1145/3511600

[B29] Liao Z. Zeng Q. Wang Q. (2015). “Chinese word pos tagging with markov logic,” in Pacific-Asia workshop on intelligence and security informatics (Ho Chi Minh City: Springer), 91–101. doi: 10.1007/978-3-319-18455-5_7

[B30] Linn Z. Z. Patil P. B. (2019). “Part of speech tagging for kayah language using hidden markov model,” in 2019 4th International conference on electrical, electronics, communication, computer technologies and optimization techniques (ICEECCOT) (Mysuru: IEEE), 228–233. doi: 10.1109/ICEECCOT46775.2019.9114552

[B31] Liu S. Best M. L. (2025). “A survey of nlp progress in sino-tibetan low-resource languages,” in Proceedings of the 2025 conference of the nations of the americas chapter of the association for computational linguistics: human language technologies (Volume 1: Long Papers) (Albuquerque, NM: ACL Anthology), 7804–7825. doi: 10.18653/v1/2025.naacl-long.396

[B32] Meelen M. Hill N. (2017). Segmenting and pos tagging classical tibetan using a memory-based tagger. Himal. Linguist. 16:2. doi: 10.5070/H916234501

[B33] Minn K. H. Soe K. M. (2019). Myanmar Word Stemming and Part-of-Speech Tagging Using Rule Based Approach (PhD thesis). MERAL Portal. University of Computer Studies, Myanmar.

[B34] Moirangthem G. Nongbri L. Singh N. J. Nongmeikapam K. (2022). “Embeddings-based parallel corpus creation for english-manipuri,” in International conference on communication and intelligent systems (Delhi: Springer), 489–502. doi: 10.1007/978-981-99-2100-3_38

[B35] Moirangthem G. Nongmeikapam K. (2021). “A back-transliteration based manipuri meetei mayek keyboard ime,” in 2021 IEEE 4th International conference on computing, power and communication technologies (GUCON) (IEEE), 1–6. doi: 10.1109/GUCON50781.2021.9573837

[B36] Myint C. (2011). “A hybrid approach for part-of-speech tagging of burmese texts,” in 2011 International conference on computer and management (CAMAN) (IEEE), 1–4. doi: 10.1109/CAMAN.2011.5778890

[B37] Neog M. Baruah N. (2023). “A deep learning framework for assamese toxic comment detection: leveraging lstm and bilstm models with attention mechanism,” in International conference on advances in data-driven computing and intelligent systems (Goa: Springer), 485–497. doi: 10.1007/978-981-99-9521-9_37

[B38] Nivre J. Marneffe M.-C. D. Ginter F. Goldberg Y. Hajic J. Manning C. D. . (2016). “Universal dependencies v1: a multilingual treebank collection,” in Proceedings of the tenth international conference on language resources and evaluation (LREC'16) (Portoroz: ACL Anthology), 1659–1666. doi: 10.63317/2hnj7od8afy8

[B39] Nongmeikapam K. Bandyopadhyay S. (2010). “Identification of MWES using CRF in Manipuri and improvement using reduplicated MWES,” in Proceedings of the international conference on natural language processing, India (Kharagpur: NLPAI).

[B40] Nongmeikapam K. Bandyopadhyay S. (2011). Genetic algorithm (ga) in feature selection for crf based manipuri multiword expression (mwe) identification. arXiv Preprint arXiv:1111.2399. doi: 10.48550/arXiv.1111.2399

[B41] Nongmeikapam K. Haobam V. Ashokumar S. Jimmy Laishram N. L. Lourembam S. (2010). “CRF based PoS Tagging of Manipur,” in Proceedings of Reso-illusion 2010, (Imphal: MIT).

[B42] Nongmeikapam K. Laishram D. Singh N. B. Chanu N. M. Bandyopadhyay S. (2011). “Identification of reduplicated multiword expressions using crf,” in International conference on intelligent text processing and computational linguistics (Tokyo: Springer), 41–51. doi: 10.1007/978-3-642-19400-9_4

[B43] Nongmeikapam K. Nonglenjaoba L. Nirmal Y. Bandyopadhyay S. (2012a). Reduplicated mwe (rmwe) helps in improving the crf based manipuri pos tagger. arXiv Preprint arXiv:1203.4933. doi: 10.48550/arXiv.1203.4933

[B44] Nongmeikapam K. Nonglenjaoba L. Roshan A. Singh T. S. Singh T. N. Bandyopadhyay S. (2012b). “Transliterated svm based manipuri pos tagging,” in Advances in computer science, engineering &applications: proceedings of the second international conference on computer science, engineering and applications (ICCSEA 2012), May 25-27, 2012, New Delhi, India, Vol. 1 (Springer), 989–999. doi: 10.1007/978-3-642-30157-5_98

[B45] Nongmeikapam K. R. K. V. R. Singh O. I. Bandyopadhyay S. (2012c). Automatic segmentation of manipuri (meiteilon) word into syllabic units. arXiv Preprint arXiv:1207.3932. doi: 10.48550/arXiv.1207.3932

[B46] Nongmeikapam K. Sharma A. U. Devi L. M. Keisam N. Singh K. D. Bandyaopadhyay S. (2012d).” Will the identification of reduplicated multiword expression (rmwe) improve the performance of svm based manipuri pos tagging?,” in *International conference on intelligent text processing and computational linguistics* (New Delhi: Springer), 117–129. doi: 10.1007/978-3-642-28604-9_10

[B47] Nongmeikapam K. Singh T. I. Chanu N. M. Bandyopadhyay S. (2014). “Manipuri chunking: an incremental model with pos and RMWE,” in Proceedings of the 11th international conference on natural language processing (Goa: NLPAI) 277–286.

[B48] Nunsanga M. V. Pakray P. Devi T. S. Singh L. L. K. (2023). Enhancing hmm-based pos tagger for mizo language. J. Intell. Fuzzy Syst. 45, 11725–11736. doi: 10.3233/JIFS-224220

[B49] Nunsanga M. V. Pakray P. Lallawmsanga C. Singh L. (2021). Part-of-speech tagging for mizo language using conditional random field. Comput. Sist. 25, 803–812. doi: 10.13053/cys-25-4-4044

[B50] Pakray P. Pal A. Majumder G. Gelbukh A. (2015). “Resource building and parts-of-speech (pos) tagging for the mizo language,” in 2015 Fourteenth Mexican international conference on artificial intelligence (MICAI) (Morelos: IEEE), 3–7. doi: 10.1109/MICAI.2015.7

[B51] Pathak D. Narzary S. Nandi S. Som B. (2025). Part-of-speech tagger for bodo language using deep learning approach. Nat. Lang. Process. 31, 215–229. doi: 10.1017/nlp.2024.15

[B52] Patra B. G. Debbarma K. Das D. Bandyopadhyay S. (2012). “Part of speech (pos) tagger for kokborok,” in Proceedings of COLING 2012: Posters (Mumbai: IIT Bombay), 923–932.

[B53] Phukan R. Neog M. Baruah N. (2023). “A deep learning based approach for spelling error detection in the assamese language,” in 2023 14th International conference on computing communication and networking technologies (ICCCNT) (Delhi: IEEE), 1–7. doi: 10.1109/ICCCNT56998.2023.10306972

[B54] Sarma S. K. Brahma B. Gogoi M. Ramchiary M. B. (2010). “A wordnet for bodo language: Structure and development,” in Global wordnet conference (GWC10), (Mumbai: GWA).

[B55] Sarma S. K. Sarmah D. Brahma B. Bharali H. Mahanta M. Saikia U. (2012). “Building multilingual lexical resources using wordnets: Structure, design and implementation,” in Proceedings of the 3rd Workshop on cognitive aspects of the Lexicon (Mumbai: IIT Bombay), 161–170.

[B56] Sarmah J. Sarma S. K. (2016a). “Survey on word sense disambiguation: an initiative towards an indo-aryan language”. in *IJEM* (Hongkong: MECS Press) 6, 37–52. doi: 10.5815/ijem.2016.03.04

[B57] Sarmah J. Sarma S. K. (2016b). “Word sense disambiguation for assamese,” in 2016 IEEE 6th international conference on advanced computing (IACC) (Bhimavaram: IEEE), 146–151. doi: 10.1109/IACC.2016.36

[B58] Sarmah J. Sarma S. K. Barman A. K. (2019). “Development of assamese rule based stemmer using wordnet,” in Proceedings of the 10th global WordNet conference (Wroclaw: GWA-ACL Anthology), 135–139. doi: 10.18653/v1/2019.gwc-1.17

[B59] Shao Y. Hardmeier C. Tiedemann J. Nivre J. (2017). “Character-based joint segmentation and pos tagging for chinese using bidirectional rnn-crf,” in Proceedings of the eighth international joint conference on natural language processing (Volume 1: Long Papers) (Taipei: ACL Anthology), 173–183.

[B60] Shen Y. Li J. Huang S. Zhou Y. Xie X. Zhao Q. (2022). “Data augmentation for low-resource word segmentation and pos tagging of ancient chinese texts,” in Proceedings of the second workshop on language technologies for historical and ancient languages (Marseille: ACL Anthology), 169–173.

[B61] Singh T. D. (2008). Manipuri pos tagging using crf and svm: A language independent approach.

[B62] Singh T. D. Bandyopadhyay S. (2008). “Morphology driven manipuri pos tagger,” in Proceedings of the IJCNLP-08 workshop on NLP for less privileged languages (Hyderabad: ACL Anthology).

[B63] Singha K. R. Purkayastha B. S. Singha K. D. (2012a). Part of speech tagging in manipuri: a rule based approach. Int. J. Comput. Applic. 51:31–36. doi: 10.5120/8111-1727

[B64] Singha K. R. Purkayastha B. S. Singha K. D. (2012b). Part of speech tagging in manipuri with hidden markov model. Int. J. Comput. Sci. Issues (IJCSI) 9:146.

[B65] Siram J. Sambyo K. Sarkar A. (2022a). “Part-of-speech (pos) tagging for the nyishi language,” in Advances in information communication technology and computing: proceedings of AICTC 2021 (Bikaner: Springer), 191–199. doi: 10.1007/978-981-19-0619-0_17

[B66] Siram J. Sambyo K. Sarkar A. (2022b). Parts of speech tagging of the nyishi language using hmm. Adv. Eng. Sci. 54, 3873–3880.

[B67] Talukdar K. Sarma S. K. (2023a). “Parts of speech taggers for indo aryan languages: a critical review of approaches and performances,” in 2023 4th International conference on computing and communication systems (I3CS) (Shillong: IEEE), 1–6. doi: 10.1109/I3CS58314.2023.10127336

[B68] Talukdar K. Sarma S. K. (2023b). “Upos tagger for low resource assamese language: LSTM and bilstm based modelling,” in 2023 IEEE International conference on machine learning and applied network technologies (ICMLANT) (El Salvador: IEEE), 1–6. doi: 10.1109/ICMLANT59547.2023.10372865

[B69] Talukdar K. Sarma S. K. Naznin F. Kashyap K. (2023). “Influence of data quality and quantity on assamese-bodo neural machine translation,” in 2023 14th International conference on computing communication and networking technologies (ICCCNT) (Delhi: IEEE), 1–5. doi: 10.1109/ICCCNT56998.2023.10306702

[B70] Wang L. Chen Z. Yang H. (2019). “Tpos tagging method based on bilstm_crf model,” in International conference of pioneering computer scientists, engineers and educators (Guilin: Springer), 490–503. doi: 10.1007/978-981-15-0118-0_38

[B71] Xiangxiu C. Qun N. Renqing N. Nyima T. Zhao Q. (2022). “Research on tibetan part-of-speech tagging based on transformer,” in 2022 3rd International conference on pattern recognition and machine learning (PRML) (Chengdu: IEEE), 315–320. doi: 10.1109/PRML56267.2022.9882234

[B72] Xu Q. Wang Z. (2022). A data-driven model for automated chinese word segmentation and pos tagging. Comput. Intell. Neurosci. 2022:7622392. doi: 10.1155/2022/7622392 36156940 PMC9507729

[B73] Xue N. Xia F. Chiou F.-D. Palmer M. (2005). The penn chinese treebank: phrase structure annotation of a large corpus. Natural Lang. Eng. 11, 207–238. doi: 10.1017/S135132490400364X

[B74] Zhang H.-P. Yu H.-K. Xiong D. Liu Q. (2003). “Hhmm-based chinese lexical analyzer ictclas,” in Proceedings of the second SIGHAN workshop on Chinese language processing (Sapporo: ACL Anthology), 184–187. doi: 10.3115/1119250.1119280

